# Transcriptional targets of amyotrophic lateral sclerosis/frontotemporal dementia protein TDP-43 – meta-analysis and interactive graphical database

**DOI:** 10.1242/dmm.049418

**Published:** 2022-09-13

**Authors:** Maize C. Cao, Emma L. Scotter

**Affiliations:** School of Biological Sciences and Centre for Brain Research, University of Auckland, 3A Symonds Street, Auckland 1010, New Zealand

**Keywords:** Amyotrophic lateral sclerosis, Frontotemporal dementia, TDP-43, RNA-seq, Loss of function, Differentially expressed genes, Exon usage

## Abstract

TDP-43 proteinopathy is the major pathology in amyotrophic lateral sclerosis (ALS) and tau-negative frontotemporal dementia (FTD). Mounting evidence implicates loss of normal TDP-43 RNA-processing function as a key pathomechanism. However, the RNA targets of TDP-43 differ by report, and have never been formally collated or compared between models and disease, hampering understanding of TDP-43 function. Here, we conducted re-analysis and meta-analysis of publicly available RNA-sequencing datasets from six TDP-43-knockdown models, and TDP-43-immunonegative neuronal nuclei from ALS/FTD brain, to identify differentially expressed genes (DEGs) and differential exon usage (DEU) events. There was little overlap in DEGs between knockdown models, but *PFKP*, *STMN2*, *CFP*, *KIAA1324* and *TRHDE* were common targets and were also differentially expressed in TDP-43-immunonegative neurons. DEG enrichment analysis revealed diverse biological pathways including immune and synaptic functions. Common DEU events in human datasets included well-known targets *POLDIP3* and *STMN2*, and novel targets *EXD3*, *MMAB*, *DLG5* and *GOSR2*. Our interactive database (https://www.scotterlab.auckland.ac.nz/research-themes/tdp43-lof-db/) allows further exploration of TDP-43 DEG and DEU targets. Together, these data identify TDP-43 targets that can be exploited therapeutically or used to validate loss-of-function processes.

This article has an associated First Person interview with the first author of the paper.

## Abstract (precis)

**Summary:** A meta-analysis and interactive database of TDP-43 target genes and splicing events identified in publicly available RNA-sequencing datasets from six TDP-43 knockdown models and TDP-43-immunonegative neurons in amyotrophic lateral sclerosis/frontotemporal dementia.

## INTRODUCTION

TDP-43 (encoded by *TARDBP*) is a predominantly nuclear DNA- and RNA-binding protein first discovered to bind to the trans-active response element in the human immunodeficiency virus (HIV)-1 sequence (Ou et al., 1995). TDP-43 was subsequently found to be the major constituent of pathogenic aggregates in amyotrophic lateral sclerosis (ALS) and frontotemporal dementia (FTD) neuropathology (Arai et al., 2006; Neumann et al., 2006). Indeed, hyper-phosphorylated and aggregated cytoplasmic TDP-43 is the pathological signature in almost all cases of ALS and ∼50% of FTD patients (Neumann et al., 2006; Ling et al., 2013; Mackenzie et al., 2007). Other neurodegenerative diseases also manifest with TDP-43 neuropathology, including Alzheimer's disease, Parkinson's disease and Huntington's disease (Amador-Ortiz et al., 2007; Arai et al., 2009; Higashi et al., 2007; James et al., 2016; McAleese et al., 2017; Nakashima-Yasuda et al., 2007; Schwab et al., 2008; Dewan et al., 2021). There is also a clear relationship between the development of neurodegenerative disease and mutations in other RNA-binding proteins that favor their aggregation (Kim et al., 2013; Vance et al., 2009; Johnson et al., 2014; Elden et al., 2010). Strikingly, the regional patterning of neuronal loss both in the brain and spinal cord closely reflects the patterning of TDP-43 aggregate deposition (Brettschneider et al., 2014, 2013; Mackenzie et al., 2013). However, TDP-43 protein inclusions represent only one species across a spectrum of conformations in which TDP-43 can exist (Scotter et al., 2014), and their ease of detection has likely influenced our perception of the pathomechanisms of disease.

**Fig. 1.**
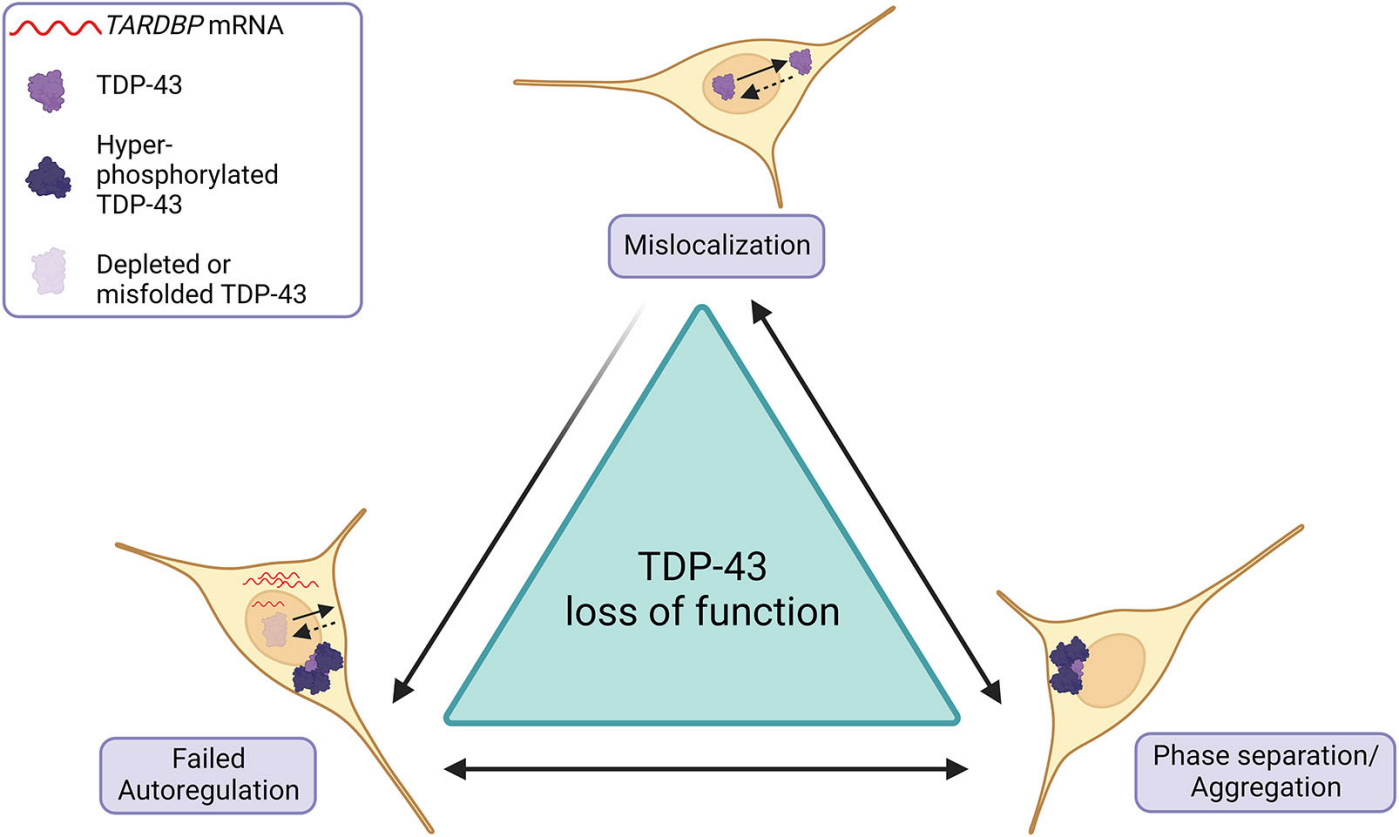
**Schematic of the mechanisms of TDP-43 loss of function in ALS.** (Top) Normally, TDP-43 is actively imported into the nucleus and passively diffuses (Pinarbasi et al., 2018), giving TDP-43 its predominantly nuclear localization. However, TDP-43 in amyotrophic lateral sclerosis (ALS) is frequently mislocalized to the cytoplasm, leading to nuclear TDP-43 depletion. (Bottom right) Cytoplasmic TDP-43 is prone to phase separation and aggregation, with hyperphosphorylated aggregates further sequestering TDP-43. (Bottom left) Readily detectable TDP-43 nuclear depletion or sequestration into aggregates, or less easily detected misfolding, can deplete the functional pool of TDP-43. TDP-43 loss of function leads to failed autoregulation of *TARDBP* (enhancing TDP-43 translation), in addition to failed regulation of myriad other TDP-43 targets, many of which are unknown or unvalidated.

The gain-of-toxic-function hypothesis for TDP-43 in ALS emerged from three key findings: (1) that almost all inherited ALS including *TARDBP*-mutant ALS is inherited dominantly (Sreedharan et al., 2008; Rutherford et al., 2008); (2) that the characteristic pathology is the appearance of cytoplasmic TDP-43 aggregates absent from non-neurodegenerative disease tissue (Arai et al., 2006; Neumann et al., 2006); and (3) that TDP-43 overexpression paradigms in animal models recapitulated these TDP-43 aggregates and the symptoms of human ALS (Wils et al., 2010; Li et al., 2010; Wegorzewska et al., 2009; Johnson et al., 2008). Recognition that loss of normal TDP-43 function may also, or instead, be pathogenic in ALS was based upon the observation that TDP-43, both in human ALS tissue and transgenic animal models harboring TDP-43 inclusions, is cleared from its normal location in the nucleus (Mitra et al., 2019; Braak and Del Tredici, 2018; Walker et al., 2015; Braak et al., 2017). Nuclear-to-cytoplasmic mislocalization of TDP-43 likely feeds into, and is further induced by, TDP-43 aggregation in the cytoplasm via a sequestration mechanism (Walker et al., 2015; Amlie-Wolf et al., 2015; Mutihac et al., 2015; Winton et al., 2008) (Fig. 1). TDP-43 fulfils specific cytoplasmic functions, including the regulation of stress granules, which are proposed by some to ‘seed’ TDP-43 aggregation (Birsa et al., 2020; Molliex et al., 2015; Fernandes et al., 2018; Becker et al., 2017). However, TDP-43 is a predominantly nuclear protein, and the majority of its RNA-processing roles are executed in the nucleus (Ou et al., 1995; Bowden and Dormann, 2016; Ling et al., 2010; Sephton et al., 2011; Buratti et al., 2001; Fiesel et al., 2012; Shiga et al., 2012; De Conti et al., 2015; Ling et al., 2015; Tollervey et al., 2011). These include regulation of alternative splicing, enhancement or repression of exon and cryptic exon inclusion, mRNA transport and polyadenylation (Ling et al., 2015; Tollervey et al., 2011; Buratti and Baralle, 2008; Buratti et al., 2004; Fallini et al., 2012; Klim et al., 2019; Melamed et al., 2019; Polymenidou et al., 2011). Nuclear TDP-43 is also able to autoregulate its own mRNA levels through a negative-feedback loop by binding its own 3′ untranslated region (UTR) (Ayala et al., 2011), so loss of TDP-43 from the nucleus likely further contributes to TDP-43 overproduction, phase separation and aggregation, and sequestration (Tziortzouda et al., 2021) (Fig. 1). Loss of appropriate TDP-43 RNA-processing function is evidenced in human ALS by extensive transcriptional change and mis-splicing (Tollervey et al., 2011; Buratti and Baralle, 2008, 2010; Polymenidou et al., 2011). Notably, similar transcriptional changes, motor neuron pathology and motor symptoms are seen both in animal models with TDP-43 inclusions and in models that are based upon TDP-43 knockdown (Mihevc et al., 2016; Broeck et al., 2014). Thus, loss of nuclear TDP-43 function is clearly critical to the pathogenesis of ALS.

In recognition of the impact of TDP-43 loss of function on gene expression in ALS and FTD, an increasing number of studies report the transcriptome-wide effect of TDP-43 depletion. Although certain TDP-43 targets, such as *RANBP1* and *POLDIP3*, are clearly reproducible in multiple studies (Fiesel et al., 2012; Shiga et al., 2012; Ling et al., 2015; Polymenidou et al., 2011; Mihevc et al., 2016; Roczniak-Ferguson and Ferguson, 2019; Stalekar et al., 2015), there has yet to be a formal analysis published of common TDP-43 loss-of-function targets. Different cell types are obviously transcriptomically unique, as are the same cell types derived from different species, meaning that the influence of TDP-43 on gene expression and splicing is context dependent (Ling et al., 2015; Jeong et al., 2017). Here, we re-analyzed publicly available RNA-sequencing (RNA-seq) datasets from TDP-43-depleted model systems, as well as a human ALS/FTD neuronal nuclei dataset demonstrating loss of nuclear TDP-43, to examine common transcriptional patterns of TDP-43 loss of function. Elucidating markers of TDP-43 loss of function will enable better understanding of disease mechanisms and the extent to which TDP-43 loss of function is associated with neurodegeneration. Further, such markers could serve as biomarkers and/or targets for treatment.

## RESULTS

### Validation of *TARDBP* depletion in TDP-43 knockdown models and in TDP-negative ALS/FTD neuronal nuclei

**Fig. 2.**
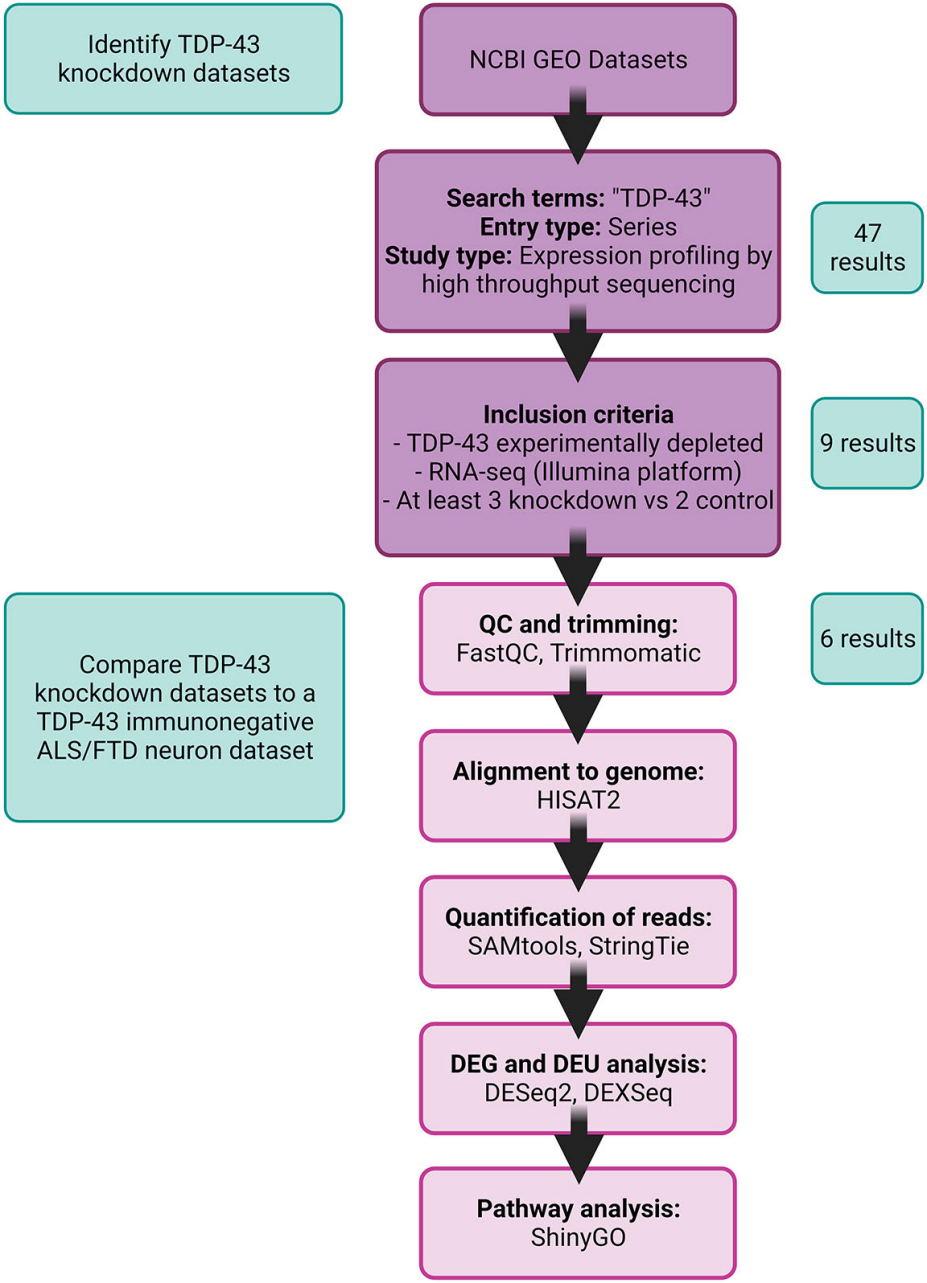
**Study selection and RNA-seq data-processing pipeline.** RNA-seq datasets were selected from the National Center for Biotechnology Information (NCBI) Gene Expression Omnibus (GEO) database using search term and study type, filtered for studies that performed RNA-seq on models with experimental TDP-43 depletion, and processed through a common bioinformatic pipeline. DEG, differentially expressed gene; DEU, differential exon usage. TDP-43 knockdown datasets were then compared with a TDP-43-immunonegative amyotrophic lateral sclerosis (ALS)/frontotemporal dementia (FTD) neuronal nuclei dataset.

Forty-seven RNA-sequencing datasets were identified using ‘TDP-43’ as a keyword. Nine studies met the inclusion criteria for re-analysis [raw data available, appropriate sample size, TDP-43 depleted experimentally (‘knockdown’)]; however, only six of these met quality control thresholds and were fully processed (Fig. 2). Of these six, three were performed on cells derived from humans {GSE136366 (human HeLa cells; Roczniak-Ferguson and Ferguson, 2019), GSE122069 (human SH-SY5Y cells; Melamed et al., 2019) and GSE121569 [human induced motor neurons (ihMNs); Klim et al., 2019)]}, two from mouse [GSE116456 (mouse mammary gland; Zhao et al., 2020) and GSE27218 (mouse striatum; Polymenidou et al., 2011)], and one from rat [GSE135611 (rat primary astrocytes; LaRocca et al., 2019)] (Table S1). An additional RNA-seq dataset was identified in which neuronal nuclei had been sorted from ALS/FTD tissue according to nuclear TDP-43 immunoreactivity [GSE126543 (cortical neuronal nuclei with or without detectable TDP-43 immunolabeling from seven ALS/FTD human brains; Liu et al., 2019)]. This dataset also met quality control thresholds and was processed using the same pipeline (Fig. 2).

### Differentially expressed genes shared by TDP-43 knockdown and TDP-negative ALS/FTD neuronal nuclei datasets

**Fig. 3.**
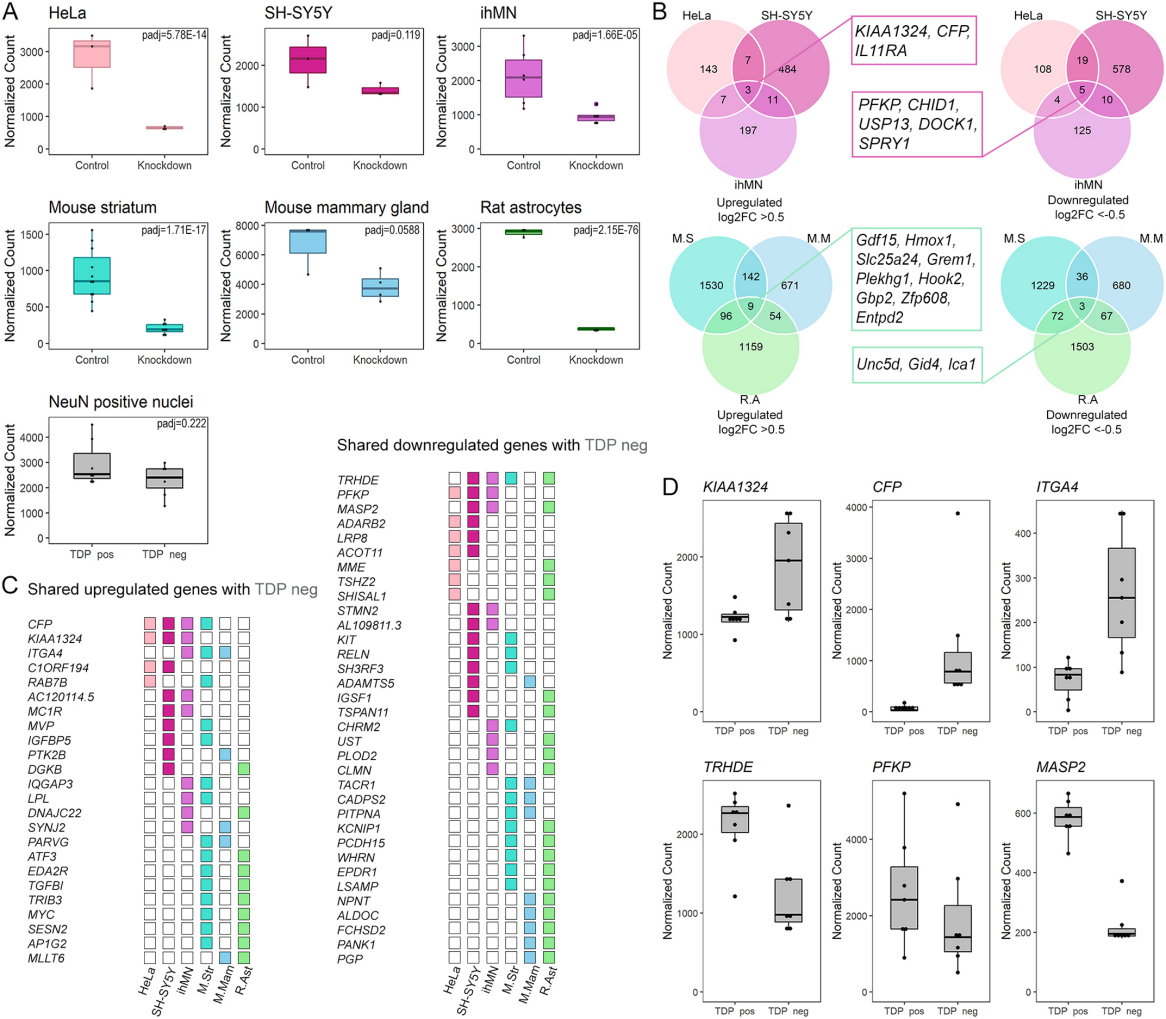
**DEGs shared by TDP-43 knockdown and TDP-negative ALS/FTD neuronal nuclei datasets were cell-type specific and species specific.** (A) Normalized counts of *TARDBP/Tardbp* transcripts from TDP-43 knockdown studies and in ALS/FTD neuronal nuclei. (B) Venn diagrams comparing upregulated and downregulated DEGs (*P*adj<0.05) among human datasets and among rodent datasets. (C) DEGs shared with the ALS/FTD TDP-43-immunonegative (TDP neg) neuronal nuclei dataset. (D) Top three upregulated and downregulated DEGs shared between the TDP-43 knockdown studies and the TDP neg neuronal nuclei dataset. ihMN, human induced motor neurons; TDP pos, TDP-43 immunopositive.

We first examined differential gene expression between control and TDP-43-knockdown samples [human HeLa cells (Roczniak-Ferguson and Ferguson, 2019), human SH-SY5Y cells (Melamed et al., 2019), ihMNs (Klim et al., 2019), mouse mammary gland (Zhao et al., 2020), mouse striatum (Polymenidou et al., 2011) and rat primary astrocytes (LaRocca et al., 2019)], and ALS/FTD TDP-43-immunopositive (TDP pos) and TDP-43-immunonegative (TDP neg) neuronal nuclei (Liu et al., 2019), using the DESeq2 package. Depletion of *TARDBP/Tardbp* was examined in HeLa, SH-SY5Y, ihMN, mouse striatum, mouse mammary gland and rat astrocytes (Fig. 3A). The extent of *TARDBP/Tardbp* depletion was greater in TDP-43 knockdown samples than in sorted ALS/FTD TDP neg neuronal nuclei, as expected given that the latter were selected for nuclear clearance of TDP-43 protein, rather than being subject to *TARDBP* knockdown. All datasets represent models of TDP-43 loss of function.

There were three upregulated differentially expressed genes (DEGs) and five downregulated DEGs shared by all human-derived TDP-43 knockdown models, and nine upregulated and three downregulated DEGs shared by all rodent-derived TDP-43 knockdown models (Fig. 3B). There was no overlap of DEGs shared among all human-derived models and DEGs shared among all rodent-derived models (Fig. 3B). Despite this, both human- and rodent-derived datasets shared DEGs with ALS/FTD TDP neg neuronal nuclei (Fig. 3C). These included known TDP-43-regulated transcripts such as *PFKP* and *STMN2* (both downregulated).

The upregulated DEGs shared by the largest number of TDP-43 knockdown models and with ALS/FTD TDP neg neuronal nuclei were *KIAA1324*, *CFP* and *ITGA4*. The shared downregulated DEGs were *TRHDE*, *PFKP* and *MASP2* (Fig. 3D). DEGs [adjusted *P*-value (*P*adj)<0.05] in each model are listed in Tables S2 and S3 (only genes with log2FC between −0.5 and >0.5), or can be explored interactively at https://www.scotterlab.auckland.ac.nz/research-themes/tdp43-lof-db/ (all significant DEGs), filtering by model system or gene of interest. Overall, DEGs were highly variable between datasets, but the sharing of DEGs between model systems validates them as conserved TDP-43 targets, and sharing of DEGs with ALS/FTD TDP neg neuronal nuclei validates those DEGs as being disease relevant.

### Altered biological processes, molecular functions and cellular components with TDP-43 knockdown

**Fig. 4.**
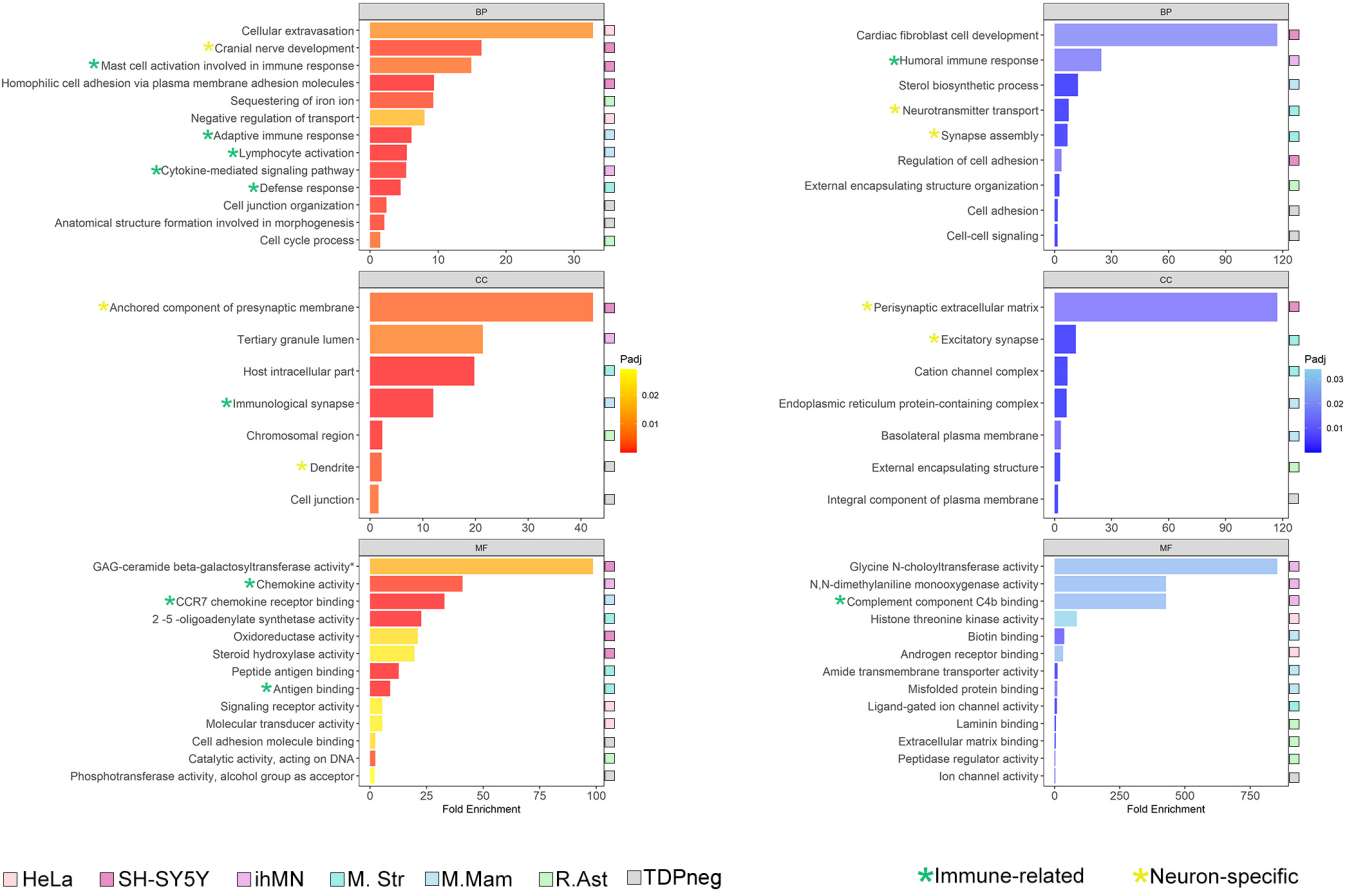
**Altered biological processes, molecular functions and cellular components with TDP-43 knockdown.** Enriched terms from gene ontology (GO) analysis (*P*adj<0.05) of upregulated genes (left) and downregulated genes (right). Terms are categorized into biological process (BP), cellular component (CC) and molecular function (MF). The model system that each term belongs to is denoted by a colored box. GO terms with shared ancestor terms are indicated by highlighting. Keys to model systems and ancestor terms shown at the bottom. ihMN, human induced motor neurons; M. Mam, mouse mammary gland; M. Str, mouse striatum; R. Ast, rat astrocytes.

Having identified certain genes with conserved patterns of regulation by TDP-43 between cell types and species, we examined the effect of TDP-43 knockdown on biological processes (BPs), molecular functions (MFs) and cellular components (CCs) using gene ontology (GO) enrichment analysis. The top three significant terms for BPs, MFs and CCs for both upregulated genes (Fig. 4, left) and downregulated genes (Fig. 4, right) from each dataset are collated in Fig. 4 and Table S4.

Two patterns emerged after redundancy was eliminated from the GO terms: many terms were related to immune response (Fig. 4, green asterisks), and many were neuron specific (Fig. 4, yellow asterisks). Mouse striatum had three BP/CC terms for downregulated genes that were neuron specific. The mouse striatum is composed of various cell types, but these data imply neuron-selective effects of TDP-43 loss of function. Supporting this, other datasets with significant neuron-specific BP or CC terms were SH-SY5Y and ALS/FTD TDP neg neuronal nuclei.

### Differential exon usage events shared by TDP-43 knockdown and TDP-negative ALS/FTD neuronal nuclei datasets

As TDP-43 is known to regulate splicing and cryptic exon expression (Ling et al., 2015; Tollervey et al., 2011), we next examined differential exon usage (DEU) between control and TDP-43-knockdown samples, and ALS/FTD TDP pos and TDP neg neuronal nuclei, using the DEXSeq package. This package quantifies changes in the relative usage of exons or parts of exons (annotated by a feature ID, e.g. E001) between conditions and generates graphical displays. DEU events in each model are listed in Table S5, or can be explored interactively at https://www.scotterlab.auckland.ac.nz/research-themes/tdp43-lof-db/, filtering for DEU as the data type.

**Fig. 5.**
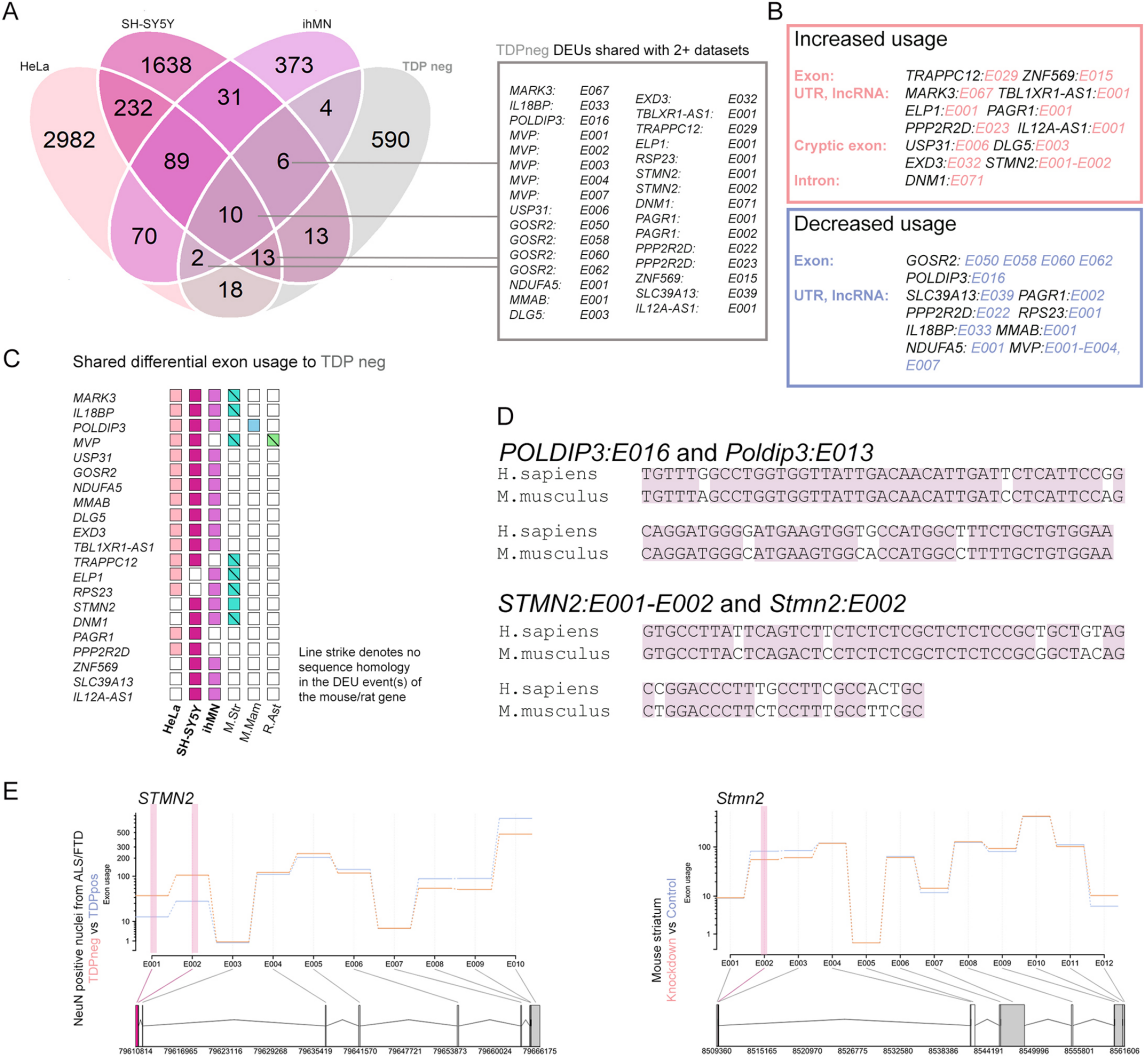
**DEU events shared by TDP-43 knockdown and TDP-negative ALS/FTD neuronal nuclei datasets.** (A) Venn diagram comparing human dataset DEU events. In the textbox are the locations of DEU events shared between ALS/FTD TDP neg neuronal nuclei and at least two other human-derived datasets. A DEU is described with a feature ID (e.g. E001) that corresponds to an exon or part of an exon. (B) DEU events according to direction of change and nature of splicing event. (C) Genes from human datasets in textbox in A showing whether a DEU was present in the orthologous mouse or rat gene (colored box), and whether the DEU region showed sequence homology between human and rodent. (D) DEU regions in *POLDIP3*/*Poldip3* and *STMN2*/*Stmn2* are homologous between human and mouse. (E) Human *STMN2* transcript (left), but not mouse *Stmn2* (right), includes a cryptic exon with TDP-43 knockdown.

There were 31 DEU events that were shared between ALS/FTD TDP neg neuronal nuclei and at least two other human-derived datasets (Fig. 5A). These DEU events included 12 within the (non-coding) 3′ UTR region of the gene. Fourteen of the 31 DEU events increased usage of an exonic element [i.e. exon inclusion, cryptic exon, intron retention, long non-coding RNA (lncRNA) upregulation], while 17 events decreased usage of the exon (i.e. exon exclusion, new intron) (Fig. 5B). For these 31 DEU events, we examined whether the orthologous mouse and rat genes in the rodent datasets demonstrated DEU events, identifying nine genes in which rodent models also showed DEU events (Fig. 5C). The differentially used exonic regions from humans were then subjected to National Center for Biotechnology Information (NCBI) Basic Local Alignment Search Tool (BLAST) analysis (https://blast.ncbi.nlm.nih.gov/Blast.cgi) using the blastn algorithm, to examine sequence homology of DEU loci between human and rodent datasets. Only two genes showed a DEU event that was shared between ALS/FTD TDP neg neuronal nuclei and a rodent dataset, and that also showed sequence homology of the region between human and mouse: *STMN2* and *POLDIP3* (Fig. 5C,D). In *POLDIP3*, exon usage in the orthologous location in both human and mouse models was decreased. However, in *STMN2*, cryptic exon usage in human models was increased, as reported previously (Klim et al., 2019; Melamed et al., 2019), but exon usage at the orthologous location in mouse was decreased (Fig. 5E).

**Fig. 6.**
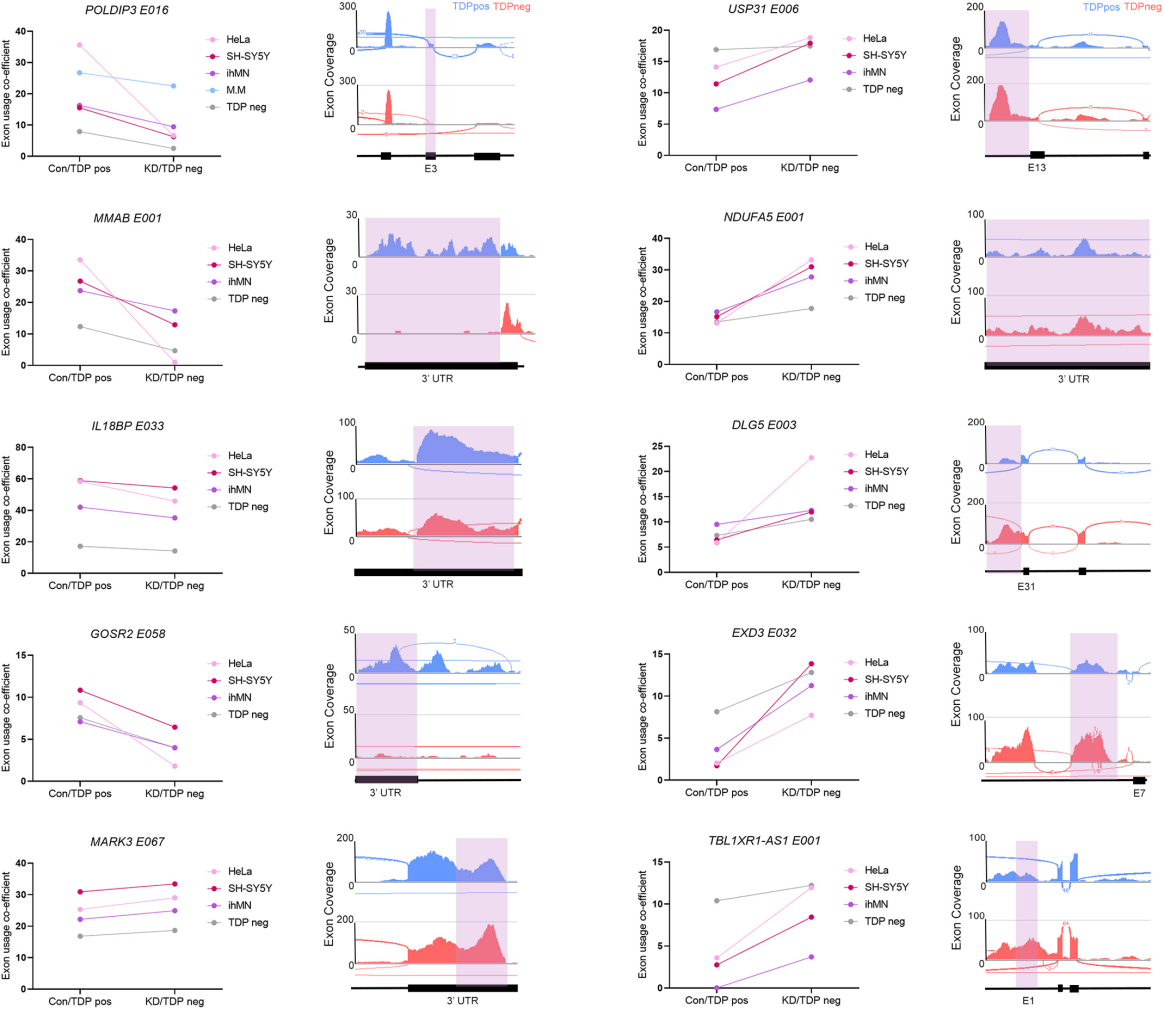
**Top shared differentially used exons – a panel of markers of TDP-43 loss of function.** The ten DEU events shared by all three human TDP-43 knockdown datasets and the ALS/FTD TDP neg dataset are showcased as a potential TDP-43 loss-of-function panel, accompanied by coverage tracks from the ALS/FTD TDP neg dataset. Of the ten shared DEU events, four were decreased with TDP-43 loss of function and six were increased.

There were ten DEU events that were shared between ALS/FTD TDP neg neuronal nuclei and all other human-derived datasets (Fig. 6), and all of these were consistent in the direction of change. Exonic element usage in *POLDIP3*, *MMAB*, *IL18BP* and *GOSR2* was decreased, and in *MARK3*, *USP31*, *NDUFA5*, *DLG5*, *EXD3* and *TBL1XR1-AS1* was increased. Of these, only *POLDIP3* showed differential usage of a coding exon, with the remainder involving changes to non-coding regions, such as introns/cryptic exons (*USP13*, *DLG5* and *EXD3*), the 3′ UTR (*MMAB*, *IL18BP*, *GOSR2*, *MARK3* and *NDUFA5*) and a lncRNA (*TBL1XR1-AS1*) (Cunningham et al., 2022). We consider these ten highly conserved DEU events to represent an ideal ‘panel’ for examining loss of TDP-43 function in humans and human models.

## DISCUSSION

As the common neuropathological feature in ALS with or without FTD, aggregated TDP-43 and its acquired toxic functions represent tractable molecular targets for therapy. Yet, increasing evidence suggests that successful therapies will also require rescue of normal physiological functions of TDP-43 that are compromised in disease. Loss of gene expression and splicing regulatory function are now widely accepted features of ALS with TDP-43 proteinopathy, but how the specific complement of *TARDBP* mRNA targets drives pathogenesis and phenotype remains unclear. Central to this is the need to identify the complement of *TARDBP* mRNA targets in distinct cell types and model systems, and to verify which of these targets is regulated by TDP-43 in human ALS/FTD neurons. We approached this problem by assessing *TARDBP* mRNA targets in human and rodent models, in neuronal and non-neuronal cell types, and identifying shared and unique targets and biological pathways under TDP-43 control. TDP-43 regulates certain common targets across myriad cell types, implying that TDP-43 proteinopathy in non-neuronal cells initiates processes that at least partially overlap with those occurring in neurons. Notably, TDP-43-dependent changes identified in multiple model systems were validated to change in human ALS/FTD neuronal nuclei with TDP-43 nuclear depletion, supporting TDP-43 loss of function as being pathomechanistic.

### DEGs as markers of TDP-43 loss of function in disease

Certain transcripts were robustly regulated by TDP-43 in multiple datasets and in human ALS/FTD neurons with nuclear TDP-43 loss. Decreased *PFKP* mRNA has been identified as a TDP-43 loss-of-function marker by several studies in addition to those in our meta-analysis (Buratti and Baralle, 2010; Coyne et al., 2021; Park et al., 2013). *PFKP* encodes the platelet isoform of phosphofructokinase, a key glycolytic enzyme that is expressed almost ubiquitously. *PFKP* is proposed to decrease under conditions of TDP-43 depletion via suppression of miR-520 (Park et al., 2013). Yet, despite the apparent promise of decreased *PFKP* as a TDP-43 loss-of-function marker, glycolysis is hypothesized to be a compensatory mechanism in ALS, and in human ALS spinal cord tissue with TDP-43 proteinopathy, *PFKP* levels were found to increase rather than decrease compared to those in controls (Manzo et al., 2019). However, TDP-43 can also bind *PFKP* directly (Tollervey et al., 2011), and *TARDBP* knockdown can result in *PFKP* cryptic exon inclusion, suggesting a direct interaction (Klim et al., 2019). Indeed, *PFKP* cryptic exon usage within intron 16 of ENSEMBL PFKP-203 was seen in all human datasets we examined except ALS/FTD TDP neg neurons (Table S5). This cryptic exon may therefore be preferable to overall *PFKP* transcript levels as a TDP-43 loss-of-function marker.

Loss of *STMN2* expression following TDP-43 depletion is associated with the emergence of a cryptic exon after exon 1, which introduces a premature polyadenylation site (Prudencio et al., 2020). This truncated *STMN2* mRNA was upregulated in the frontal cortex in FTD with TDP-43 pathology (Prudencio et al., 2020). STMN2 protein is essential for microtubule stability and thus cytoskeletal transport, synapse maintenance and homeostasis of motor neurons. STMN2 has been identified as a key TDP-43 target in neurodegeneration, with deficits in axonal outgrowth and repair following TDP-43 depletion being rescued by restoration of STMN2 alone (Klim et al., 2019; Melamed et al., 2019). Cytoskeletal dynamics are further implicated in the pathogenesis of ALS/FTD by disease-causing mutations in *DCTN1* (Münch et al., 2005), *PFN1* (Wu et al., 2012; Smith et al., 2015) and *TUBA4A* (Smith et al., 2014). Interestingly, ALS/FTD linked to any of these genes is associated with TDP-43 cytoplasmic aggregation (and thus presumably TDP-43 nuclear clearing), raising the possibility of a feed-forward interaction between TDP-43 loss of function and axonal cytoskeleton dysfunction. Indeed, a therapeutic that induces STMN2 for ALS is soon to enter clinical trial. We predict that both the *STMN2* cryptic exon and overall *STMN2* transcript levels will be of high utility in detecting neuronal subtypes with TDP-43 loss of function.

Two other shared downregulated genes among TDP-43 knockdown models and the ALS/FTD TDP neg dataset were *TRHDE* and *MASP2*. *TRHDE* (encoding thyrotropin-releasing hormone-degrading enzyme) is an M1 family metallopeptidase enriched in brain regions (Torres et al., 1986). Its main substrate is thyrotropin-releasing hormone (TRH), secreted by neurons, and, accordingly, *TRHDE* was downregulated in brain-derived or neuron-like model datasets. However, a dual TRH-mimic/TRHDE inhibitor compound reduced motor decline and spinal cord neurodegeneration in a SOD1 mouse model (Kelly et al., 2015), so loss of TRH degradation downstream of TDP-43 dysfunction is unlikely to be pathomechanistic. Also downregulated is *MASP2*, which is involved in activation of the complement system (Kidmose et al., 2012). The *MASP2* gene is immediately downstream of *TARDBP*, and thus a degree of co-regulation is expected due to similar chromatin states (Arnone et al., 2012), but these data suggest that *MASP2* levels are responsive to TDP-43 protein levels independent of chromatin packing. As is true of other genes, whether *MASP2* gene expression is useful as a TDP-43 loss-of-function marker depends on whether TDP-43 is a major determinant of its transcript levels.

Upregulated genes shared between various TDP-43 depletion datasets and ALS/FTD TDP neg neurons were *KIAA1324*, *CFP* and *ITGA4*. *KIAA1324* encodes a multifunctional protein and is also known as estrogen-induced gene 121 (*EIG121*) and endosome/lysosome-associated apoptosis and autophagy regulator (*ELAPOR1*) in humans (Deng et al., 2010), or *Elapor1* or insulin inhibitory receptor (*Iir*) in mice (Ansarullah et al., 2021). *CFP* encodes the complement factor properdin, which regulates the alternative complement pathway (Pedersen et al., 2017) and may be upregulated by chronic cell stress. *ITGA4* encodes integrin subunit alpha 4, which is overexpressed in nerve injury (Bas et al., 2015; Xing et al., 2017), promotes immune cell infiltration and inhibited therapeutically in multiple sclerosis (Butzkueven et al., 2014). As *ITGA4* is positively regulated by the lncRNA *NEAT1* (Asadi et al., 2021), and *NEAT1* binding scaffolds the phase separation of TDP-43 (Tollervey et al., 2011; Wang et al., 2020), TDP-43 loss of function might liberate *NEAT1* and thus increase *ITGA4* levels. However, *ITGA4* is also upregulated in *SOD1*- and *FUS*-mutant induced pluripotent stem cell models, which do not exhibit TDP-43 proteinopathy or dysfunction (Ziff et al., 2022). As all of these upregulated genes are sensitive to apoptosis and neuroinflammation, any upregulation specifically due to loss of TDP-43 function may be difficult to disentangle.

In general, the value of using overall expression levels of TDP-43-regulated transcripts as loss-of-function markers depends upon the following: (1) the cell types in which the transcript is expressed, and whether those cell types are subject to TDP-43 dysfunction in the tissue sampled; (2) whether the transcript is subject to secondary regulation, aside from regulation by TDP-43; and (3) whether regulation by TDP-43 is direct or indirect via other mediators also subject to change. Overall, the ability of DEGs to report upon TDP-43 function is likely to differ across different transcriptomic profiles (cell states, cell types, species).

### DEU events as markers of TDP-43 loss of function in disease

TDP-43 mainly binds non-coding stretches of DNA/RNA, such as introns, untranslated regions, intergenic regions and lncRNAs (Tollervey et al., 2011). Of the ten DEU events identified as common between the human-derived datasets (Fig. 6), only one involved a coding exon (*POLDIP3*). The skipping of exon 3 in *POLDIP3* is a frequently reproduced marker in TDP-43 knockdown studies (Fiesel et al., 2012; Shiga et al., 2012; Tollervey et al., 2011; Mihevc et al., 2016; Roczniak-Ferguson and Ferguson, 2019; Colombrita et al., 2015; Suzuki et al., 2015), in which it undergoes an isoform switch. In the absence of TDP-43, the canonical variant 1 is decreased whereas variant 2 is increased due to exon 3 exclusion (Fiesel et al., 2012; Shiga et al., 2012). Interestingly, overexpression of TDP-43 mutants in HEK293T cells also caused *POLDIP3* exon 3 exclusion, suggesting that mutants can induce loss of function of endogenous wild-type TDP-43 (Chen et al., 2019). POLDIP3 protein [also known as S6K1 Aly/REF-like target (SKAR)] interacts with exon junctional complexes to increase the translation efficiency of spliced mRNAs (Ma et al., 2008). Our validation of *POLDIP3* exon 3 exclusion in human ALS/FTD neuronal nuclei with loss of nuclear TDP-43 strongly supports *POLDIP3* as a TDP-43 loss-of-function marker in ALS tissue, and indeed increased *POLDIP3* variant 2 mRNA is seen in various motor regions of the CNS in ALS (Shiga et al., 2012).

Half of the DEU events common to all human datasets were modifiers of 3′ UTR usage. The 3′ UTR is an important regulatory region, of mRNA stability (Meijlink et al., 1985), localization (Macdonald and Struhl, 1988) and translation (Miller and Madras, 2002), and even protein–protein interactions (Berkovits and Mayr, 2015). Trans-acting factors such as RNA-binding proteins that interact with the 3′ UTR also determine its function (Mayr, 2017), meaning that altered 3′ UTR sequences may have variable effects depending on the transcriptome. Three intron retention or cryptic exon emergence events were common to all human datasets, and these can be associated with disease (Dhir and Buratti, 2010). Because changes in intron/cryptic exon usage with TDP-43 loss of function were sometimes subtle, these genes should be examined together and ideally as part of the panel of ten DEU events identified in Fig. 6. This panel may be used in the design of probes for *in situ* hybridization (RNAScope, BaseScope) of ALS/FTD tissue, in conjunction with cell-type-specific immunohistochemical markers, to identify additional cell types with TDP-43 loss of function. This panel of DEU events could also act as primers/probes for quantitative RT-PCR to assess the fidelity of ALS models or the restoration of TDP-43 function by therapeutics.

Together, the transcripts identified in the panel cover a range of biological functions, including translation (*POLDIP3*) (Ma et al., 2008), mitochondrial function (*MMAB*, *NDUFA5*) (Dobson et al., 2002; Loeffen et al., 1998), endosomal trafficking (*GOSR2*) (Lowe et al., 1997), immune response regulation (*IL18BP*) (Novick et al., 1999), microtubule regulation (*MARK3*, *DLG5*) (Gu et al., 2013; Wang et al., 2014), exonuclease activity (*EXD3*) (Gaudet et al., 2011) and deubiquitylation (*USP31*) (Lockhart et al., 2004). How the processing, translation and protein interactions of these transcripts are changed with DEU warrants future investigation. So too does the upregulation of the lncRNA *TBL1XR1-AS1*, which occurred in the absence of gene expression changes to its target transcript *TBL1XR1* in any human dataset. These DEU events represent a conserved set of markers of TDP-43 loss of function, demonstrate the promiscuity of TDP-43 effects and mirror the diversity in biological functions implicated in ALS pathogenesis.

### Models for identifying TDP-43 targets – strengths and limitations

Several of our data suggest our DESeq2 and DEXSeq analyses to be conservative methods for identifying DEGs and DEU events, and comparative studies of DEG packages have also demonstrated DESeq2 to err on the conservative side (Li et al., 2020; Soneson and Delorenzi, 2013). It is essential to identify consistent and reliable markers of TDP-43 loss of function to nominate targets with diagnostic or therapeutic potential. DEXSeq was chosen for this study for its conservative approach to controlling Type I error, leading to fewer false positives (Anders et al., 2012). TDP-43 may therefore regulate additional splicing events than those described here, and their identification could be aided by the combined use of multiple exon usage and splicing analysis tools. Conversely, exon usage changes identified in this study are unlikely to be due to sample variance and are indeed TDP-43 dependent.

In addition to the methodology used, the transcriptional targets of TDP-43 that we identified were dependent upon the species and fidelity of the cellular and animal models of ALS employed. Cryptic exons that emerge in human models of TDP-43 loss of function are not recapitulated in mouse models (Ling et al., 2015; Humphrey et al., 2017), including a cryptic exon identified in *STMN2* that has attracted significant attention in ALS/FTD research (Klim et al., 2019). Our results build upon the emerging consensus that TDP-43 has a distinct set of molecular targets in different cell types and species (Jeong et al., 2017). Human-derived transcriptomes are likely to be most suitable for identifying molecular pathways and drug targets relevant to human ALS.

The mechanism of modelling ALS is equally critical to identifying disease-relevant TDP-43 targets and pathways. Depleting TDP-43 is gaining acceptance in a field initially predominated by overexpression and TDP-43-mutant models (Wegorzewska and Baloh, 2011; Liu et al., 2013), and here we demonstrate that TDP-43 depletion recapitulates at least some of the transcriptional effects of loss of nuclear TDP-43 in ALS/FTD neuronal nuclei. TDP-43 knockdown can thus be considered an appropriate, even if partial, experimental paradigm of disease for identifying mechanisms, biomarkers and therapeutic targets.

This study highlights mRNA transcripts and parts of transcripts for which expression can robustly report upon TDP-43 loss of function. TDP-43 knockdown largely alters different biological pathways in human and rodent model systems, and human-derived models better recapitulate specific transcriptional and splicing changes that occur in ALS/FTD neuronal nuclei. Our findings enable future identification of non-neuronal cell types with TDP-43 loss of function, while revealing key players in the selective neuronal cell death that occurs in ALS and FTD.

## MATERIALS AND METHODS

### Identification of TDP-43 knockdown studies for meta-analysis

A repository search was conducted in September 2020 to identify TDP-43 knockdown studies with available RNA-seq data using the Gene Expression Omnibus (GEO) from the NCBI (http://ncbi.nlm.nih.gov/geo). The search was performed using the keyword ‘TDP-43’, and the results were filtered by setting Entry Type as ‘Series’ to capture all potential samples that belonged to a common study, and setting Study Type as ‘Expression profiling by high throughput sequencing’. Inclusion criteria for re-analyzing these datasets were as follows: (1) raw RNA-seq data available; (2) at least three TDP-43 knockdown samples and two appropriate control samples; (3) experimental depletion of TDP-43. In addition, an RNA-seq dataset was identified in which neuronal nuclei had been sorted from ALS/FTD tissue according to nuclear TDP-43 immunoreactivity; either TDP pos (normal nuclear TDP-43) or TDP neg (no nuclear TDP-43) (Liu et al., 2019) (Fig. 2). This was considered an appropriate ‘disease validation’ dataset for targets identified through meta-analysis of TDP-43 knockdown studies, because the within-case comparison of TDP pos and TDP neg neuronal nuclei paralleled the paradigm of TDP-43 loss of function by knockdown.

### Data processing and statistical analysis

The data processing pipeline is shown in Fig. 2. Raw data were downloaded from NCBI using Sequence Read Archive (SRA) Toolkit v2.9.6 (http://www.ncbi.nlm.nih.gov/sra), and quality control was applied using FastQC v0.11.9 software (https://www.bioinformatics.babraham.ac.uk/projects/fastqc/). Details of each dataset including study design, GEO accession number and library information are supplied in Table S1. Code is deposited at https://github.com/mcao051/TDP_LOF. Raw data that still contained adapter sequence content were trimmed with Trimmomatic v0.39 (Bolger et al., 2014). If fewer than 5 million reads survived trimming, the samples were not included for further analysis, and if this reduced total number of controls or knockdown samples to below the inclusion criteria, the study was excluded. Reads were then aligned to the appropriate reference genome with HISAT2 v2.2.1 and quantified with StringTie v1.3.5 (Pertea et al., 2016). Reference genome builds used were GRCh38, GRCm39 and Rnor6.0 for human, mouse and rat, respectively. Count data were imported using R package tximport (Soneson et al., 2015) to perform differential expression analysis using DESeq2 (Love et al., 2014) and DEU analysis using DEXSeq (Anders et al., 2012). DEU is a more general measure than alternative splicing, as differing exon boundaries between reads are accounted for, therefore revealing differential usage of parts of exons or introns (exonic elements) (Anders et al., 2012). DESeq2 *P*-values were calculated using Wald tests and corrected for multiple testing using the Benjamini–Hochberg method. DEU events with *P*adj<0.05 were considered significantly changed. Genes with *P*adj<0.05 were used for interactive graphical reports but only genes with log2FC between −0.5 and 0.5 were compared between models. ShinyGO v0.75 (http://bioinformatics.sdstate.edu/go/) was used for GO enrichment analysis using genes with *P*adj<0.05 and log2FC of either <−0.5 or >0.5. The genes detected after low-count filtering for each dataset were used as the background gene list. Redundant GO terms were eliminated using the ReViGO tool available at http://revigo.irb.hr/.

Interactive graphical reports were generated from the packages Glimma (Su et al., 2017) and DEXSeq (Anders et al., 2012). These can be accessed and explored further at https://www.scotterlab.auckland.ac.nz/research-themes/tdp43-lof-db/. They enable the reader to visualize and interact with all differential gene expression and exon usage analyses described herein, including genes of interest not highlighted in our study.

### Data visualization

Data were visualized using R software with ggplot2 (https://ggplot2.tidyverse.org/), ggvenn (https://CRAN.R-project.org/package=ggvenn), the plotCounts function from within DESeq2 (Love et al., 2014), DEXSeq (https://www.r-project.org/), Prism 9.0 software (GraphPad Software, La Jolla, CA, USA) and Integrative Genomics Viewer (Robinson et al., 2011). Adobe Photoshop 2021 v22.5.1 (Adobe Inc.) was used as a graphic editor.

## Supplementary Material

Supplementary information

## References

[DMM049418C1] Amador-Ortiz, C., Lin, W.-L., Ahmed, Z., Personett, D., Davies, P., Duara, R., Graff-Radford, N. R., Hutton, M. L. and Dickson, D. W. (2007). TDP-43 immunoreactivity in hippocampal sclerosis and Alzheimer's disease. *Ann. Neurol.* 61, 435-445. 10.1002/ana.21154 17469117 PMC2677204

[DMM049418C2] Amlie-Wolf, A., Ryvkin, P., Tong, R., Dragomir, I., Suh, E. R., Xu, Y., Van Deerlin, V. M., Gregory, B. D., Kwong, L. K., Trojanowski, J. Q. et al. (2015). Transcriptomic changes due to cytoplasmic TDP-43 expression reveal dysregulation of histone transcripts and nuclear chromatin. *PLoS ONE* 10, e0141836. 10.1371/journal.pone.0141836 26510133 PMC4624943

[DMM049418C3] Anders, S., Reyes, A. and Huber, W. (2012). Detecting differential usage of exons from RNA-seq data. *Genome Res.* 22, 2008-2017. 10.1101/gr.133744.111 22722343 PMC3460195

[DMM049418C4] Ansarullah, W., Jain, C., Far, F. F., Homberg, S., Wissmiller, K., Von Hahn, F. G., Raducanu, A., Schirge, S., Sterr, M., Bilekova, S. et al. (2021). Inceptor counteracts insulin signalling in beta-cells to control glycaemia. *Nature* 590, 326-331. 10.1038/s41586-021-03225-8 33505018

[DMM049418C5] Arai, T., Hasegawa, M., Akiyama, H., Ikeda, K., Nonaka, T., Mori, H., Mann, D., Tsuchiya, K., Yoshida, M., Hashizume, Y. et al. (2006). TDP-43 is a component of ubiquitin-positive tau-negative inclusions in frontotemporal lobar degeneration and amyotrophic lateral sclerosis. *Biochem. Biophys. Res. Commun.* 351, 602-611. 10.1016/j.bbrc.2006.10.093 17084815

[DMM049418C6] Arai, T., Mackenzie, I. R. A., Hasegawa, M., Nonoka, T., Niizato, K., Tsuchiya, K., Iritani, S., Onaya, M. and Akiyama, H. (2009). Phosphorylated TDP-43 in Alzheimer's disease and dementia with Lewy bodies. *Acta Neuropathol.* 117, 125-136. 10.1007/s00401-008-0480-1 19139911

[DMM049418C7] Arnone, J. T., Robbins-Pianka, A., Arace, J. R., Kass-Gergi, S. and Mcalear, M. A. (2012). The adjacent positioning of co-regulated gene pairs is widely conserved across eukaryotes. *BMC Genomics* 13, 546. 10.1186/1471-2164-13-546 23051624 PMC3500266

[DMM049418C8] Asadi, G., Rezaei Varmaziar, F., Karimi, M., Rajabinejad, M., Ranjbar, S., Gorgin Karaji, A., Salari, F., Afshar Hezarkhani, L. and Rezaiemanesh, A. (2021). Determination of the transcriptional level of long non-coding RNA NEAT-1, downstream target microRNAs, and genes targeted by microRNAs in diabetic neuropathy patients. *Immunol. Lett.* 232, 20-26. 10.1016/j.imlet.2021.01.007 33508370

[DMM049418C9] Ayala, Y. M., De Conti, L., Avendaño-Vázquez, S. E., Dhir, A., Romano, M., D'Ambrogio, A., Tollervey, J., Ule, J., Baralle, M., Buratti, E. et al. (2011). TDP-43 regulates its mRNA levels through a negative feedback loop. *EMBO J.* 30, 277-288. 10.1038/emboj.2010.310 21131904 PMC3025456

[DMM049418C10] Bas, E., Goncalves, S., Adams, M., Dinh, C. T., Bas, J. M. and Van De Water, T. R. (2015). Spiral ganglion cells and macrophages initiate neuro-inflammation and scarring following cochlear implantation. *Front. Cell Neurosci.* 9, 303. 10.3389/fncel.2015.00303 26321909 PMC4532929

[DMM049418C11] Becker, L. A., Huang, B., Bieri, G., Ma, R., Knowles, D. A., Jafar-Nejad, P., Messing, J., Kim, H. J., Soriano, A., Auburger, G. et al. (2017). Therapeutic reduction of ataxin-2 extends lifespan and reduces pathology in TDP-43 mice. *Nature* 544, 367-371. 10.1038/nature22038 28405022 PMC5642042

[DMM049418C12] Berkovits, B. D. and Mayr, C. (2015). Alternative 3′ UTRs act as scaffolds to regulate membrane protein localization. *Nature* 522, 363-367. 10.1038/nature14321 25896326 PMC4697748

[DMM049418C13] Birsa, N., Bentham, M. P. and Fratta, P. (eds). (2020). Cytoplasmic functions of TDP-43 and FUS and their role in ALS. *Semin. Cell Dev. Biol.* 99, 193-201. 10.1016/j.semcdb.2019.05.023 31132467

[DMM049418C14] Bolger, A. M., Lohse, M. and Usadel, B. (2014). Trimmomatic: a flexible trimmer for Illumina sequence data. *Bioinformatics* 30, 2114-2120. 10.1093/bioinformatics/btu170 24695404 PMC4103590

[DMM049418C15] Bowden, H. A. and Dormann, D. (2016). Altered mRNP granule dynamics in FTLD pathogenesis. *J. Neurochem.* 138 Suppl. 1, 112-133. 10.1111/jnc.13601 26938019

[DMM049418C16] Braak, H. and Del Tredici, K. (2018). Anterior cingulate cortex TDP-43 pathology in sporadic amyotrophic lateral sclerosis. *J. Neuropathol. Exp. Neurol.* 77, 74-83. 10.1093/jnen/nlx104 29186496

[DMM049418C17] Braak, H., Ludolph, A. C., Neumann, M., Ravits, J. and Del Tredici, K. (2017). Pathological TDP-43 changes in Betz cells differ from those in bulbar and spinal α-motoneurons in sporadic amyotrophic lateral sclerosis. *Acta Neuropathol.* 133, 79-90. 10.1007/s00401-016-1633-2 27757524 PMC5209403

[DMM049418C18] Brettschneider, J., Del Tredici, K., Toledo, J. B., Robinson, J. L., Irwin, D. J., Grossman, M., Suh, E. R., Van Deerlin, V. M., Wood, E. M., Baek, Y. et al. (2013). Stages of pTDP-43 pathology in amyotrophic lateral sclerosis. *Ann. Neurol.* 74, 20-38. 10.1002/ana.23937 23686809 PMC3785076

[DMM049418C19] Brettschneider, J., Arai, K., Del Tredici, K., Toledo, J. B., Robinson, J. L., Lee, E. B., Kuwabara, S., Shibuya, K., Irwin, D. J., Fang, L. et al. (2014). TDP-43 pathology and neuronal loss in amyotrophic lateral sclerosis spinal cord. *Acta Neuropathol.* 128, 423-437. 10.1007/s00401-014-1299-6 24916269 PMC4384652

[DMM049418C20] Broeck, L. V., Callaerts, P. and Dermaut, B. (2014). TDP-43-mediated neurodegeneration: towards a loss-of-function hypothesis? *Trends Mol. Med.* 20, 66-71. 10.1016/j.molmed.2013.11.003 24355761

[DMM049418C21] Buratti, E. and Baralle, F. E. (2008). Multiple roles of TDP-43 in gene expression, splicing regulation, and human disease. *Front. Biosci.* 13, 867-878. 10.2741/2727 17981595

[DMM049418C22] Buratti, E. and Baralle, F. E. (2010). The multiple roles of TDP-43 in pre-mRNA processing and gene expression regulation. *RNA Biol.* 7, 420-429. 10.4161/rna.7.4.12205 20639693

[DMM049418C23] Buratti, E., Dork, T., Zuccato, E., Pagani, F., Romano, M. and Baralle, F. E. (2001). Nuclear factor TDP-43 and SR proteins promote in vitro and in vivo CFTR exon 9 skipping. *EMBO J.* 20, 1774-1784. 10.1093/emboj/20.7.1774 11285240 PMC145463

[DMM049418C24] Buratti, E., Brindisi, A., Pagani, F. and Baralle, F. E. (2004). Nuclear factor TDP-43 binds to the polymorphic TG repeats in CFTR intron 8 and causes skipping of exon 9: a functional link with disease penetrance. *Am. J. Hum. Genet.* 74, 1322-1325. 10.1086/420978 15195661 PMC1182100

[DMM049418C25] Butzkueven, H., Kappos, L., Pellegrini, F., Trojano, M., Wiendl, H., Patel, R. N., Zhang, A., Hotermans, C. and Belachew, S. (2014). Efficacy and safety of natalizumab in multiple sclerosis: interim observational programme results. *J. Neurol. Neurosurg. Psychiatry* 85, 1190-1197. 10.1136/jnnp-2013-306936 24532785 PMC4215289

[DMM049418C26] Chen, H.-J., Topp, S. D., Hui, H. S., Zacco, E., Katarya, M., Mcloughlin, C., King, A., Smith, B. N., Troakes, C., Pastore, A. et al. (2019). RRM adjacent TARDBP mutations disrupt RNA binding and enhance TDP-43 proteinopathy. *Brain* 142, 3753-3770. 10.1093/brain/awz313 31605140 PMC6885686

[DMM049418C27] Colombrita, C., Onesto, E., Buratti, E., De La Grange, P., Gumina, V., Baralle, F. E., Silani, V. and Ratti, A. (2015). From transcriptomic to protein level changes in TDP-43 and FUS loss-of-function cell models. *Biochim. Biophys. Acta* 1849, 1398-1410. 10.1016/j.bbagrm.2015.10.015 26514432

[DMM049418C28] Coyne, A. N., Baskerville, V., Zaepfel, B. L., Dickson, D. W., Rigo, F., Bennett, F., Lusk, C. P. and Rothstein, J. D. (2021). Nuclear accumulation of CHMP7 initiates nuclear pore complex injury and subsequent TDP-43 dysfunction in sporadic and familial ALS. *Sci. Transl. Med.* 13, eabe1923. 10.1126/scitranslmed.abe1923 34321318 PMC9022198

[DMM049418C29] Cunningham, F., Allen, J. E., Allen, J., Alvarez-Jarreta, J., Amode, M. R., Armean, I. M., Austine-Orimoloye, O., Azov, A. G., Barnes, I., Bennett, R. et al. (2022). Ensembl 2022. *Nucleic Acids Res.* 50, D988-DD95. 10.1093/nar/gkab1049 34791404 PMC8728283

[DMM049418C30] De Conti, L., Akinyi, M. V., Mendoza-Maldonado, R., Romano, M., Baralle, M. and Buratti, E. (2015). TDP-43 affects splicing profiles and isoform production of genes involved in the apoptotic and mitotic cellular pathways. *Nucleic Acids Res.* 43, 8990-9005. 10.1093/nar/gkv814 26261209 PMC4605304

[DMM049418C31] Deng, L., Feng, J. and Broaddus, R. R. (2010). The novel estrogen-induced gene EIG121 regulates autophagy and promotes cell survival under stress. *Cell Death Dis.* 1, e32. 10.1038/cddis.2010.9 21072319 PMC2976047

[DMM049418C32] Dewan, R., Chia, R., Ding, J., Hickman, R. A., Stein, T. D., Abramzon, Y., Ahmed, S., Sabir, M. S., Portley, M. K., Tucci, A. et al. (2021). Pathogenic Huntingtin repeat expansions in patients with frontotemporal dementia and amyotrophic lateral sclerosis. *Neuron* 109, 448-60.e4. 10.1016/j.neuron.2020.11.005 33242422 PMC7864894

[DMM049418C33] Dhir, A. and Buratti, E. (2010). Alternative splicing: role of pseudoexons in human disease and potential therapeutic strategies. *FEBS J.* 277, 841-855. 10.1111/j.1742-4658.2009.07520.x 20082636

[DMM049418C34] Dobson, C. M., Wai, T., Leclerc, D., Kadir, H., Narang, M. and Lerner-Ellis, J. P. (2002). Identification of the gene responsible for the cblB complementation group of vitamin B12-dependent methylmalonic aciduria. *Hum. Mol. Genet.* 11, 3361-3369. 10.1093/hmg/11.26.3361 12471062

[DMM049418C35] Elden, A. C., Kim, H.-J., Hart, M. P., Chen-Plotkin, A. S., Johnson, B. S., Fang, X., Armakola, M., Geser, F., Greene, R., Lu, M. M. et al. (2010). Ataxin-2 intermediate-length polyglutamine expansions are associated with increased risk for ALS. *Nature* 466, 1069-1075. 10.1038/nature09320 20740007 PMC2965417

[DMM049418C36] Fallini, C., Bassell, G. J. and Rossoll, W. (2012). The ALS disease protein TDP-43 is actively transported in motor neuron axons and regulates axon outgrowth. *Hum. Mol. Genet.* 21, 3703-3718. 10.1093/hmg/dds205 22641816 PMC3406762

[DMM049418C37] Fernandes, N., Eshleman, N. and Buchan, J. R. (2018). Stress granules and ALS: a case of causation or correlation? *Adv. Neurobiol.* 20, 173-212. 10.1007/978-3-319-89689-2_7 29916020

[DMM049418C38] Fiesel, F. C., Weber, S. S., Supper, J., Zell, A. and Kahle, P. J. (2012). TDP-43 regulates global translational yield by splicing of exon junction complex component SKAR. *Nucleic Acids Res.* 40, 2668-2682. 10.1093/nar/gkr1082 22121224 PMC3315294

[DMM049418C39] Gaudet, P., Livstone, M. S., Lewis, S. E. and Thomas, P. D. (2011). Phylogenetic-based propagation of functional annotations within the Gene Ontology consortium. *Brief. Bioinform.* 12, 449-462. 10.1093/bib/bbr042 21873635 PMC3178059

[DMM049418C40] Gu, G. J., Lund, H., Wu, D., Blokzijl, A., Classon, C., Von Euler, G., Landegren, U., Sunnemark, D. and Kamali-Moghaddam, M. (2013). Role of individual MARK isoforms in phosphorylation of tau at Ser(2)(6)(2) in Alzheimer's disease. *Neuromolecular Med.* 15, 458-469. 10.1007/s12017-013-8232-3 23666762

[DMM049418C41] Higashi, S., Iseki, E., Yamamoto, R., Minegishi, M., Hino, H., Fujisawa, K., Togo, T., Katsuse, O., Uchikado, H., Furukawa, Y. et al. (2007). Concurrence of TDP-43, tau and α-synuclein pathology in brains of Alzheimer's disease and dementia with Lewy bodies. *Brain Res.* 1184, 284-294. 10.1016/j.brainres.2007.09.048 17963732

[DMM049418C42] Humphrey, J., Emmett, W., Fratta, P., Isaacs, A. M. and Plagnol, V. (2017). Quantitative analysis of cryptic splicing associated with TDP-43 depletion. *BMC Med Genomics* 10, 38. 10.1186/s12920-017-0274-1 28549443 PMC5446763

[DMM049418C43] James, B. D., Wilson, R. S., Boyle, P. A., Trojanowski, J. Q., Bennett, D. A. and Schneider, J. A. (2016). TDP-43 stage, mixed pathologies, and clinical Alzheimer's-type dementia. *Brain* 139, 2983-2993. 10.1093/brain/aww224 27694152 PMC5091047

[DMM049418C44] Jeong, Y. H., Ling, J. P., Lin, S. Z., Donde, A. N., Braunstein, K. E., Majounie, E., Traynor, B. J., LaClair, K. D., Lloyd, T. E. and Wong, P. C. et al. (2017). Tdp-43 cryptic exons are highly variable between cell types. *Mol. Neurodegener.* 12, 1-9. 10.1186/s13024-016-0144-x 28153034 PMC5289002

[DMM049418C45] Johnson, B. S., Mccaffery, J. M., Lindquist, S. and Gitler, A. D. (2008). A yeast TDP-43 proteinopathy model: exploring the molecular determinants of TDP-43 aggregation and cellular toxicity. *Proc. Natl. Acad. Sci. USA* 105, 6439-6444. 10.1073/pnas.0802082105 18434538 PMC2359814

[DMM049418C46] Johnson, J. O., Pioro, E. P., Boehringer, A., Chia, R., Feit, H., Renton, A. E., Pliner, H. A., Abramzon, Y., Marangi, G., Winborn, B. J. et al. (2014). Mutations in the Matrin 3 gene cause familial amyotrophic lateral sclerosis. *Nat. Neurosci.* 17, 664-666. 10.1038/nn.3688 24686783 PMC4000579

[DMM049418C47] Kelly, J. A., Boyle, N. T., Cole, N., Slator, G. R., Colivicchi, M. A., Stefanini, C., Gobbo, O. L., Scalabrino, G. A., Ryan, S. M., Elamin, M. et al. (2015). First-in-class thyrotropin-releasing hormone (TRH)-based compound binds to a pharmacologically distinct TRH receptor subtype in human brain and is effective in neurodegenerative models. *Neuropharmacology* 89, 193-203. 10.1016/j.neuropharm.2014.09.024 25281210

[DMM049418C48] Kidmose, R. T., Laursen, N. S., Dobó, J., Kjaer, T. R., Sirotkina, S., Yatime, L., Sottrup-Jensen, L., Thiel, S., Gál, P. and Andersen, G. R. (2012). Structural basis for activation of the complement system by component C4 cleavage. *Proc. Natl. Acad. Sci. USA* 109, 15425-15430. 10.1073/pnas.1208031109 22949645 PMC3458355

[DMM049418C49] Kim, H. J., Kim, N. C., Wang, Y.-D., Scarborough, E. A., Moore, J., Diaz, Z., Maclea, K. S., Freibaum, B., Li, S., Molliex, A. et al. (2013). Mutations in prion-like domains in hnRNPA2B1 and hnRNPA1 cause multisystem proteinopathy and ALS. *Nature* 495, 467-473. 10.1038/nature11922 23455423 PMC3756911

[DMM049418C50] Klim, J. R., Williams, L. A., Limone, F., Guerra San Juan, I., Davis-Dusenbery, B. N., Mordes, D. A., Burberry, A., Steinbaugh, M. J., Gamage, K. K., Kirchner, R. et al. (2019). ALS-implicated protein TDP-43 sustains levels of STMN2, a mediator of motor neuron growth and repair. *Nat. Neurosci.* 22, 167-179. 10.1038/s41593-018-0300-4 30643292 PMC7153761

[DMM049418C51] Larocca, T. J., Mariani, A., Watkins, L. R. and Link, C. D. (2019). TDP-43 knockdown causes innate immune activation via protein kinase R in astrocytes. *Neurobiol. Dis.* 132, 104514. 10.1016/j.nbd.2019.104514 31229690 PMC6834892

[DMM049418C52] Li, Y., Ray, P., Rao, E. J., Shi, C., Guo, W., Chen, X., Woodruff, E. A., Fushimi, K. and Wu, J. Y. (2010). A Drosophila model for TDP-43 proteinopathy. *Proc. Natl. Acad. Sci. USA* 107, 3169-3174. 10.1073/pnas.0913602107 20133767 PMC2840283

[DMM049418C53] Li, X., Cooper, N. G. F., O'toole, T. E. and Rouchka, E. C. (2020). Choice of library size normalization and statistical methods for differential gene expression analysis in balanced two-group comparisons for RNA-seq studies. *BMC Genomics* 21, 75. 10.1186/s12864-020-6502-7 31992223 PMC6986029

[DMM049418C54] Ling, S.-C., Albuquerque, C. P., Han, J. S., Lagier-Tourenne, C., Tokunaga, S., Zhou, H. and Cleveland, D. W. (2010). ALS-associated mutations in TDP-43 increase its stability and promote TDP-43 complexes with FUS/TLS. *Proc. Natl. Acad. Sci. USA* 107, 13318-13323. 10.1073/pnas.1008227107 20624952 PMC2922163

[DMM049418C55] Ling, S.-C., Polymenidou, M. and Cleveland, D. W. (2013). Converging mechanisms in ALS and FTD: disrupted RNA and protein homeostasis. *Neuron* 79, 416-438. 10.1016/j.neuron.2013.07.033 23931993 PMC4411085

[DMM049418C56] Ling, J. P., Pletnikova, O., Troncoso, J. C. and Wong, P. C. (2015). TDP-43 repression of nonconserved cryptic exons is compromised in ALS-FTD. *Science* 349, 650-655. 10.1126/science.aab0983 26250685 PMC4825810

[DMM049418C57] Liu, Y.-C., Chiang, P.-M. and Tsai, K.-J. (2013). Disease animal models of TDP-43 proteinopathy and their pre-clinical applications. *Int. J. Mol. Sci.* 14, 20079-20111. 10.3390/ijms141020079 24113586 PMC3821604

[DMM049418C58] Liu, E. Y., Russ, J., Cali, C. P., Phan, J. M., Amlie-Wolf, A. and Lee, E. B. (2019). Loss of nuclear TDP-43 is associated with decondensation of LINE retrotransposons. *Cell Rep.* 27, 1409-21.e6. 10.1016/j.celrep.2019.04.003 31042469 PMC6508629

[DMM049418C59] Lockhart, P. J., Hulihan, M., Lincoln, S., Hussey, J., Skipper, L., Bisceglio, G., Wilkes, K. and Farrer, M. J. (2004). Identification of the human ubiquitin specific protease 31 (USP31) gene: structure, sequence and expression analysis. *DNA Seq.* 15, 9-14. 10.1080/10855660310001638197 15354349

[DMM049418C60] Loeffen, J. L. C. M., Triepels, R. H., Van Den Heuvel, L. P., Schuelke, M., Buskens, C. A. F., Smeets, R. J. P., Trijbels, J. M. F. and Smeitink, J. A. M. (1998). cDNA of eight nuclear encoded subunits of NADH:ubiquinone oxidoreductase: human complex I cDNA characterization completed. *Biochem. Biophys. Res. Commun.* 253, 415-422. 10.1006/bbrc.1998.9786 9878551

[DMM049418C61] Love, M. I., Huber, W. and Anders, S. (2014). Moderated estimation of fold change and dispersion for RNA-seq data with DESeq2. *Genome Biol.* 15, 550. 10.1186/s13059-014-0550-8 25516281 PMC4302049

[DMM049418C62] Lowe, S. L., Peter, F., Subramaniam, V. N., Wong, S. H. and Hong, W. (1997). A SNARE involved in protein transport through the Golgi apparatus. *Nature* 389, 881-884. 10.1038/39923 9349823

[DMM049418C63] Ma, X. M., Yoon, S.-O., Richardson, C. J., Jülich, K. and Blenis, J. (2008). SKAR links pre-mRNA splicing to mTOR/S6K1-mediated enhanced translation efficiency of spliced mRNAs. *Cell* 133, 303-313. 10.1016/j.cell.2008.02.031 18423201

[DMM049418C64] Macdonald, P. M. and Struhl, G. (1988). cis-acting sequences responsible for anterior localization of bicoid mRNA in Drosophila embryos. *Nature* 336, 595-598. 10.1038/336595a0 3143913

[DMM049418C65] Mackenzie, I. R. A., Bigio, E. H., Ince, P. G., Geser, F., Neumann, M., Cairns, N. J., Kwong, L. K., Forman, M. S., Ravits, J., Stewart, H. et al. (2007). Pathological TDP-43 distinguishes sporadic amyotrophic lateral sclerosis from amyotrophic lateral sclerosis with SOD1 mutations. *Ann. Neurol.* 61, 427-434. 10.1002/ana.21147 17469116

[DMM049418C66] Mackenzie, I. R., Arzberger, T., Kremmer, E., Troost, D., Lorenzl, S., Mori, K., Weng, S.-M., Haass, C., Kretzschmar, H. A., Edbauer, D. et al. (2013). Dipeptide repeat protein pathology in C9ORF72 mutation cases: clinico-pathological correlations. *Acta Neuropathol.* 126, 859-879. 10.1007/s00401-013-1181-y 24096617

[DMM049418C67] Manzo, E., Lorenzini, I., Barrameda, D., O'Conner, A. G., Barrows, J. M., Starr, A., Kovalik, T., Rabichow, B. E., Lehmkuhl, E. M., Shreiner, D. D. et al. (2019). Glycolysis upregulation is neuroprotective as a compensatory mechanism in ALS. *eLife* 8, e45114. 10.7554/eLife.45114 31180318 PMC6557627

[DMM049418C68] Mayr, C. (2017). Regulation by 3′-untranslated regions. *Annu. Rev. Genet.* 51, 171-194. 10.1146/annurev-genet-120116-024704 28853924

[DMM049418C69] Mcaleese, K. E., Walker, L., Erskine, D., Thomas, A. J., Mckeith, I. G. and Attems, J. J. B. (2017). TDP-43 pathology in Alzheimer's disease, dementia with Lewy bodies and ageing. *Brain Pathol.* 27, 472-479. 10.1111/bpa.12424 27495267 PMC8029292

[DMM049418C70] Meijlink, F., Curran, T., Miller, A. D. and Verma, I. M. (1985). Removal of a 67-base-pair sequence in the noncoding region of protooncogene fos converts it to a transforming gene. *Proc. Natl. Acad. Sci. USA* 82, 4987-4991. 10.1073/pnas.82.15.4987 2991903 PMC390483

[DMM049418C71] Melamed, Z., López-Erauskin, J., Baughn, M. W., Zhang, O., Drenner, K., Sun, Y., Freyermuth, F., McMahon, M. A., Beccari, M. S., Artates, J. W. et al. (2019). Premature polyadenylation-mediated loss of stathmin-2 is a hallmark of TDP-43-dependent neurodegeneration. *Nat. Neurosci.* 22, 180-190. 10.1038/s41593-018-0293-z 30643298 PMC6348009

[DMM049418C72] Mihevc, S. P., Baralle, M., Buratti, E. and Rogelj, B. (2016). TDP-43 aggregation mirrors TDP-43 knockdown, affecting the expression levels of a common set of proteins. *Sci. Rep.* 6, 33996. 10.1038/srep33996 27665936 PMC5036055

[DMM049418C73] Miller, G. M. and Madras, B. K. (2002). Polymorphisms in the 3′-untranslated region of human and monkey dopamine transporter genes affect reporter gene expression. *Mol. Psychiatry* 7, 44-55. 10.1038/sj.mp.4000921 11803445

[DMM049418C74] Mitra, J., Guerrero, E. N., Hegde, P. M., Liachko, N. F., Wang, H., Vasquez, V., Gao, J., Pandey, A., Taylor, J. P., Kraemer, B. C. et al. (2019). Motor neuron disease-associated loss of nuclear TDP-43 is linked to DNA double-strand break repair defects. *Proc. Natl. Acad. Sci. USA* 116, 4696-4705. 10.1073/pnas.1818415116 30770445 PMC6410842

[DMM049418C75] Molliex, A., Temirov, J., Lee, J., Coughlin, M., Kanagaraj, A. P., Kim, H. J., Mittag, T. and Taylor, J. P. (2015). Phase separation by low complexity domains promotes stress granule assembly and drives pathological fibrillization. *Cell* 163, 123-133. 10.1016/j.cell.2015.09.015 26406374 PMC5149108

[DMM049418C76] Münch, C., Rosenbohm, A., Sperfeld, A.-D., Uttner, I., Reske, S., Krause, B. J., Sedlmeier, R., Meyer, T., Hanemann, C. O., Stumm, G. et al. (2005). Heterozygous R1101K mutation of the DCTN1 gene in a family with ALS and FTD. *Ann. Neurol.* 58, 777-780. 10.1002/ana.20631 16240349

[DMM049418C77] Mutihac, R., Alegre-Abarrategui, J., Gordon, D., Farrimond, L., Yamasaki-Mann, M., Talbot, K. and Wade-Martins, R. (2015). TARDBP pathogenic mutations increase cytoplasmic translocation of TDP-43 and cause reduction of endoplasmic reticulum Ca(2)(+) signaling in motor neurons. *Neurobiol. Dis.* 75, 64-77. 10.1016/j.nbd.2014.12.010 25526708

[DMM049418C78] Nakashima-Yasuda, H., Uryu, K., Robinson, J., Xie, S. X., Hurtig, H., Duda, J. E., Arnold, S. E., Siderowf, A., Grossman, M., Leverenz, J. B. et al. (2007). Co-morbidity of TDP-43 proteinopathy in Lewy body related diseases. *Acta Neuropathol.* 114, 221-229. 10.1007/s00401-007-0261-2 17653732

[DMM049418C79] Neumann, M., Sampathu, D. M., Kwong, L. K., Truax, A. C., Micsenyi, M. C., Chou, T. T., Bruce, J., Schuck, T., Grossman, M., Clark, C. M. et al. (2006). Ubiquitinated TDP-43 in frontotemporal lobar degeneration and amyotrophic lateral sclerosis. *Science* 314, 130-133. 10.1126/science.1134108 17023659

[DMM049418C80] Novick, D., Kim, S.-H., Fantuzzi, G., Reznikov, L. L., Dinarello, C. A. and Rubinstein, M. (1999). Interleukin-18 binding protein: a novel modulator of the Th1 cytokine response. *Immunity* 10, 127-136. 10.1016/S1074-7613(00)80013-8 10023777

[DMM049418C81] Ou, S. H., Wu, F., Harrich, D., García-Martínez, L. F. and Gaynor, R. B. (1995). Cloning and characterization of a novel cellular protein, TDP-43, that binds to human immunodeficiency virus type 1 TAR DNA sequence motifs. *J. Virol.* 69, 3584-3596. 10.1128/jvi.69.6.3584-3596.1995 7745706 PMC189073

[DMM049418C82] Park, Y.-Y., Kim, S.-B., Han, H. D., Sohn, B. H., Kim, J. H., Liang, J., Lu, Y., Rodriguez-Aguayo, C., Lopez-Berestein, G., Mills, G. B. et al. (2013). Tat-activating regulatory DNA-binding protein regulates glycolysis in hepatocellular carcinoma by regulating the platelet isoform of phosphofructokinase through microRNA 520. *J. Hepatol.* 58, 182-191. 10.1002/hep.26310 PMC3923572 23389994

[DMM049418C83] Pedersen, D. V., Roumenina, L., Jensen, R. K., Gadeberg, T. A. F., Marinozzi, C., Picard, C., Rybkine, T., Thiel, S., Sørensen, U. B. S., Stover, C. et al. (2017). Functional and structural insight into properdin control of complement alternative pathway amplification. *EMBO J.* 36, 1084-1099. 10.15252/embj.201696173 28264884 PMC5391138

[DMM049418C84] Pertea, M., Kim, D., Pertea, G. M., Leek, J. T. and Salzberg, S. L. (2016). Transcript-level expression analysis of RNA-seq experiments with HISAT, StringTie and Ballgown. *Nat. Protoc.* 11, 1650-1667. 10.1038/nprot.2016.095 27560171 PMC5032908

[DMM049418C85] Pinarbasi, E. S., Cağatay, T., Fung, H. Y. J., Li, Y. C., Chook, Y. M. and Thomas, P. J. (2018). Active nuclear import and passive nuclear export are the primary determinants of TDP-43 localization. *Sci. Rep.* 8, 7083. 10.1038/s41598-018-25008-4 29728608 PMC5935693

[DMM049418C86] Polymenidou, M., Lagier-Tourenne, C., Hutt, K. R., Huelga, S. C., Moran, J., Liang, T. Y., Ling, S.-C., Sun, E., Wancewicz, E., Mazur, C. et al. (2011). Long pre-mRNA depletion and RNA missplicing contribute to neuronal vulnerability from loss of TDP-43. *Nat. Neurosci.* 14, 459-468. 10.1038/nn.2779 21358643 PMC3094729

[DMM049418C87] Prudencio, M., Humphrey, J., Pickles, S., Brown, A.-L., Hill, S. E., Kachergus, J. M., Shi, J., Heckman, M. G., Spiegel, M. R., Cook, C. et al. (2020). Truncated stathmin-2 is a marker of TDP-43 pathology in frontotemporal dementia. *J. Clin. Invest.* 130, 6080-6092. 10.1172/JCI139741 32790644 PMC7598060

[DMM049418C88] Robinson, J. T., Thorvaldsdóttir, H., Winckler, W., Guttman, M., Lander, E. S., Getz, G. and Mesirov, J. P. (2011). Integrative genomics viewer. *Nat. Biotechnol.* 29, 24-26. 10.1038/nbt.1754 21221095 PMC3346182

[DMM049418C89] Roczniak-Ferguson, A. and Ferguson, S. M. (2019). Pleiotropic requirements for human TDP-43 in the regulation of cell and organelle homeostasis. *Life Sci. Alliance* 2, e201900358. 10.26508/lsa.201900358 31527135 PMC6749094

[DMM049418C90] Rutherford, N. J., Zhang, Y.-J., Baker, M., Gass, J. M., Finch, N. C. A., Xu, Y.-F., Stewart, H., Kelley, B. J., Kuntz, K., Crook, R. J. P. et al. (2008). Novel mutations in TARDBP (TDP-43) in patients with familial amyotrophic lateral sclerosis. *PLoS Genet.* 4, e1000193. 10.1371/journal.pgen.1000193 18802454 PMC2527686

[DMM049418C91] Schwab, C., Arai, T., Hasegawa, M., Yu, S. and Mcgeer, P. L. (2008). Colocalization of transactivation-responsive DNA-binding protein 43 and huntingtin in inclusions of Huntington disease. *J. Neuropathol. Exp. Neurol.* 67, 1159-1165. 10.1097/NEN.0b013e31818e8951 19018245

[DMM049418C92] Scotter, E. L., Vance, C., Nishimura, A. L., Lee, Y.-B., Chen, H.-J., Urwin, H., Sardone, V., Mitchell, J. C., Rogelj, B., Rubinsztein, D. C. et al. (2014). Differential roles of the ubiquitin proteasome system and autophagy in the clearance of soluble and aggregated TDP-43 species. *J. Cell Sci.* 127, 1263-1278. 10.1242/jcs.140087 24424030 PMC3953816

[DMM049418C93] Sephton, C. F., Cenik, C., Kucukural, A., Dammer, E. B., Cenik, B., Han, Y. H., Dewey, C. M., Roth, F. P., Herz, J., Peng, J. et al. (2011). Identification of neuronal RNA targets of TDP-43-containing ribonucleoprotein complexes. *J. Biol. Chem.* 286, 1204-1215. 10.1074/jbc.M110.190884 21051541 PMC3020728

[DMM049418C94] Shiga, A., Ishihara, T., Miyashita, A., Kuwabara, M., Kato, T., Watanabe, N., Yamahira, A., Kondo, C., Yokoseki, A., Takahashi, M. et al. (2012). Alteration of POLDIP3 splicing associated with loss of function of TDP-43 in tissues affected with ALS. *PLoS ONE* 7, e43120. 10.1371/journal.pone.0043120 22900096 PMC3416794

[DMM049418C95] Smith, B. N., Ticozzi, N., Fallini, C., Gkazi, A. S., Topp, S., Kenna, K. P., Scotter, E. L., Kost, J., Keagle, P., Miller, J. W. et al. (2014). Exome-wide rare variant analysis identifies TUBA4A mutations associated with familial ALS. *Neuron* 84, 324-331. 10.1016/j.neuron.2014.09.027 25374358 PMC4521390

[DMM049418C96] Smith, B. N., Vance, C., Scotter, E. L., Troakes, C., Wong, C. H., Topp, S., Maekawa, S., King, A. and Mitchell, J. C. (2015). Novel mutations support a role for Profilin 1 in the pathogenesis of ALS. *Neurobiol. Aging* 36, 1602.e17-27. 10.1016/j.neurobiolaging.2014.10.032 PMC4357530 25499087

[DMM049418C97] Soneson, C. and Delorenzi, M. (2013). A comparison of methods for differential expression analysis of RNA-seq data. *BMC Bioinformatics* 14, 91. 10.1186/1471-2105-14-91 23497356 PMC3608160

[DMM049418C98] Soneson, C., Love, M. I. and Robinson, M. D. (2015). Differential analyses for RNA-seq: transcript-level estimates improve gene-level inferences. *F1000Res.* 4, 1521. 10.12688/f1000research.7563.1 26925227 PMC4712774

[DMM049418C99] Sreedharan, J., Blair, I. P., Tripathi, V. B., Hu, X., Vance, C., Rogelj, B., Ackerley, S., Durnall, J. C., Williams, K. L., Buratti, E. et al. (2008). TDP-43 mutations in familial and sporadic amyotrophic lateral sclerosis. *Science* 319, 1668-1672. 10.1126/science.1154584 18309045 PMC7116650

[DMM049418C100] Stalekar, M., Yin, X., Rebolj, K., Darovic, S., Troakes, C., Mayr, M., Shaw, C. E. and Rogelj, B. (2015). Proteomic analyses reveal that loss of TDP-43 affects RNA processing and intracellular transport. *Neuroscience* 293, 157-170. 10.1016/j.neuroscience.2015.02.046 25743254

[DMM049418C101] Su, S., Law, C. W., Ah-Cann, C., Asselin-Labat, M.-L., Blewitt, M. E. and Ritchie, M. E. (2017). Glimma: interactive graphics for gene expression analysis. *Bioinformatics* 33, 2050-2052. 10.1093/bioinformatics/btx094 28203714 PMC5870845

[DMM049418C102] Suzuki, H., Shibagaki, Y., Hattori, S. and Matsuoka, M. (2015). Nuclear TDP-43 causes neuronal toxicity by escaping from the inhibitory regulation by hnRNPs. *Hum. Mol. Genet.* 24, 1513-1527. 10.1093/hmg/ddu563 25378556

[DMM049418C103] Tollervey, J. R., Curk, T., Rogelj, B., Briese, M., Cereda, M., Kayikci, M., König, J., Hortobágyi, T., Nishimura, A. L., Župunski, V. et al. (2011). Characterizing the RNA targets and position-dependent splicing regulation by TDP-43. *Nat. Neurosci.* 14, 452-458. 10.1038/nn.2778 21358640 PMC3108889

[DMM049418C104] Torres, H., Charli, J. L., Gonzalez-Noriega, A., Vargas, M. A. and Joseph-Bravo, P. (1986). Subcellular distribution of the enzymes degrading thyrotropin releasing hormone and metabolites in rat brain. *Neurochem. Int.* 9, 103-110. 10.1016/0197-0186(86)90038-0 20493107

[DMM049418C105] Tziortzouda, P., Van Den Bosch, L. and Hirth, F. (2021). Triad of TDP43 control in neurodegeneration: autoregulation, localization and aggregation. *Nat. Rev. Neurosci.* 22, 197-208. 10.1038/s41583-021-00431-1 33654312

[DMM049418C106] Vance, C., Rogelj, B., Hortobágyi, T., De Vos, K. J., Nishimura, A. L., Sreedharan, J., Hu, X., Smith, B., Ruddy, D., Wright, P. et al. (2009). Mutations in FUS, an RNA processing protein, cause familial amyotrophic lateral sclerosis type 6. *Science* 323, 1208-1211. 10.1126/science.1165942 19251628 PMC4516382

[DMM049418C107] Walker, A. K., Spiller, K. J., Ge, G., Zheng, A., Xu, Y., Zhou, M., Tripathy, K., Kwong, L. K., Trojanowski, J. Q. and Lee, V. M.-Y. (2015). Functional recovery in new mouse models of ALS/FTLD after clearance of pathological cytoplasmic TDP-43. *Acta Neuropathol.* 130, 643-660. 10.1007/s00401-015-1460-x 26197969 PMC5127391

[DMM049418C108] Wang, S.-H. J., Celic, I., Choi, S.-Y., Riccomagno, M., Wang, Q., Sun, L. O., Mitchell, S. P., Vasioukhin, V., Huganir, R. L. and Kolodkin, A. L. (2014). Dlg5 regulates dendritic spine formation and synaptogenesis by controlling subcellular N-cadherin localization. *J. Neurosci.* 34, 12745-12761. 10.1523/JNEUROSCI.1280-14.2014 25232112 PMC4166160

[DMM049418C109] Wang, C., Duan, Y., Duan, G., Wang, Q., Zhang, K., Deng, X., Qian, B., Gu, J., Ma, Z., Zhang, S. et al. (2020). Stress induces dynamic, cytotoxicity-antagonizing TDP-43 nuclear bodies via paraspeckle LncRNA NEAT1-mediated liquid-liquid phase separation. *Mol. Cell* 79, 443-58.e7. 10.1016/j.molcel.2020.06.019 32649883

[DMM049418C110] Wegorzewska, I. and Baloh, R. H. (2011). TDP-43-based animal models of neurodegeneration: new insights into ALS pathology and pathophysiology. *Neurodegener. Dis.* 8, 262-274. 10.1159/000321547 21124004 PMC3214943

[DMM049418C111] Wegorzewska, I., Bell, S., Cairns, N. J., Miller, T. M. and Baloh, R. H. (2009). TDP-43 mutant transgenic mice develop features of ALS and frontotemporal lobar degeneration. *Proc. Natl. Acad. Sci. USA* 106, 18809-18814. 10.1073/pnas.0908767106 19833869 PMC2762420

[DMM049418C112] Wils, H., Kleinberger, G., Janssens, J., Pereson, S., Joris, G., Cuijt, I., Smits, V., Ceuterick-De Groote, C., Van Broeckhoven, C. and Kumar-Singh, S. (2010). TDP-43 transgenic mice develop spastic paralysis and neuronal inclusions characteristic of ALS and frontotemporal lobar degeneration. *Proc. Natl. Acad. Sci. USA* 107, 3858-3863. 10.1073/pnas.0912417107 20133711 PMC2840518

[DMM049418C113] Winton, M. J., Igaz, L. M., Wong, M. M., Kwong, L. K., Trojanowski, J. Q. and Lee, V. M.-Y. (2008). Disturbance of nuclear and cytoplasmic TAR DNA-binding protein (TDP-43) induces disease-like redistribution, sequestration, and aggregate formation. *J. Biol. Chem.* 283, 13302-13309. 10.1074/jbc.M800342200 18305110 PMC2442318

[DMM049418C114] Wu, C.-H., Fallini, C., Ticozzi, N., Keagle, P. J., Sapp, P. C., Piotrowska, K., Lowe, P., Koppers, M., Mckenna-Yasek, D., Baron, D. M. et al. (2012). Mutations in the profilin 1 gene cause familial amyotrophic lateral sclerosis. *Nature* 488, 499-503. 10.1038/nature11280 22801503 PMC3575525

[DMM049418C115] Xing, L., Cheng, Q., Zha, G. and Yi, S. (2017). Transcriptional profiling at high temporal resolution reveals robust immune/inflammatory responses during rat sciatic nerve recovery. *Mediators Inflamm.* 2017, 3827841. 10.1155/2017/3827841 28490837 PMC5405595

[DMM049418C116] Zhao, L., Ke, H., Xu, H., Wang, G.-D., Zhang, H., Zou, L., Xiang, S., Li, M., Peng, L., Zhou, M. et al. (2020). TDP-43 facilitates milk lipid secretion by post-transcriptional regulation of Btn1a1 and Xdh. *Nat. Commun..* 11, 341. 10.1038/s41467-019-14183-1 31953403 PMC6969145

[DMM049418C117] Ziff, O. J., Clarke, B. E., Taha, D. M., Crerar, H., Luscombe, N. M. and Patani, R. (2022). Meta-analysis of human and mouse ALS astrocytes reveals multi-omic signatures of inflammatory reactive states. *Genome Res.* 32, 71-84. 10.1101/gr.275939.121 34963663 PMC8744676

