# Supplementary material for: Transcriptional targets of amyotrophic lateral sclerosis/frontotemporal dementia protein TDP-43 – meta-analysis and interactive graphical database
Source: Dis Model Mech. 2022 Sep 13;15(9):dmm049418. doi: 10.1242/dmm.049418 (PMC9509890; doi:10.1242/dmm.049418)
Supplement: Supplementary information [file dmm-15-049418-s1.pdf]

Table S1. Details of RNA-seq datasets used in this study

Click here to download Table S1

Table S2. Downregulated DEGs from all datasets (padj <0.05, log2FC between -0.5 and >0.5)

Click here to download Table S2

Table S3. Upregulated DEGs from all datasets (padj <0.05, log2FC between -0.5 and >0.5)

Click here to download Table S3

Table S4. Top three significant GO terms (biological process, molecular function, cellular component) from each dataset

Click here to download Table S4

Table S5. Differential exon usage events from all datasets (padj < 0.05)

Click here to download Table S5
